# EBP2, a novel NPM‐ALK‐interacting protein in the nucleolus, contributes to the proliferation of ALCL cells by regulating tumor suppressor p53

**DOI:** 10.1002/1878-0261.12822

**Published:** 2020-11-19

**Authors:** Yuki Uchihara, Kenji Tago, Hiroomi Tamura, Megumi Funakoshi‐Tago

**Affiliations:** ^1^ Division of Hygienic Chemistry Faculty of Pharmacy Keio University Tokyo Japan; ^2^ Division of Structural Biochemistry Department of Biochemistry Jichi Medical University Shimotsuke‐shi Japan

**Keywords:** Akt, EBP2, mTORC1, NPM‐ALK, nucleolus, p53

## Abstract

The oncogenic fusion protein nucleophosmin‐anaplastic lymphoma kinase (NPM‐ALK), found in anaplastic large‐cell lymphoma (ALCL), localizes to the cytosol, nucleoplasm, and nucleolus. However, the relationship between its localization and transforming activity remains unclear. We herein demonstrated that NPM‐ALK localized to the nucleolus by binding to nucleophosmin 1 (NPM1), a nucleolar protein that exhibits shuttling activity between the nucleolus and cytoplasm, in a manner that was dependent on its kinase activity. In the nucleolus, NPM‐ALK interacted with Epstein–Barr virus nuclear antigen 1‐binding protein 2 (EBP2), which is involved in rRNA biosynthesis. Moreover, enforced expression of NPM‐ALK induced tyrosine phosphorylation of EBP2. Knockdown of EBP2 promoted the activation of the tumor suppressor p53, leading to G_0_/G_1_‐phase cell cycle arrest in Ba/F3 cells transformed by NPM‐ALK and ALCL patient‐derived Ki‐JK cells, but not ALCL patient‐derived SUDH‐L1 cells harboring p53 gene mutation. In Ba/F3 cells transformed by NPM‐ALK and Ki‐JK cells, p53 activation induced by knockdown of EBP2 was significantly inhibited by Akt inhibitor GDC‐0068, mTORC1 inhibitor rapamycin, and knockdown of Raptor, an essential component of mTORC1. These results suggest that the knockdown of EBP2 triggered p53 activation through the Akt‐mTORC1 pathway in NPM‐ALK‐positive cells. Collectively, the present results revealed the critical repressive mechanism of p53 activity by EBP2 and provide a novel therapeutic strategy for the treatment of ALCL.

## Graphical abstract

Nucleophosmin‐anaplastic lymphoma kinase (NPM‐ALK) inactivates tumor suppressor p53 by negatively regulating the activation of the Akt‐mTORC1 pathway through an interaction with EBP2 in the nucleolus in anaplastic large‐cell lymphoma cells.

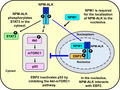

**Abbreviations**
ALCL: anaplastic large‐cell lymphoma
EBP2: EBNA1‐binding protein 2
IMT: inflammatory myofibroblastic tumors
mTOR: mechanistic target of rapamycin
mTORC1: mTOR complex 1
NoLS: nucleolar localization signal
NPM1: nucleophosmin 1
NPM‐ALK: nucleophosmin‐anaplastic lymphoma kinase
NSCLC: non‐small cell lung cancer
TPM3: tropomyosin 3

## 1 Introduction

Anaplastic lymphoma kinase (ALK) is a receptor tyrosine kinase, which is a member of the insulin receptor superfamily, and contains an extracellular ligand‐binding domain, transmembrane domain, and intracellular tyrosine kinase domain [1]. The translocation of chromosomes including the *ALK* gene has been demonstrated in various cancers including anaplastic large‐cell lymphoma (ALCL), non‐small cell lung cancer (NSCLC), and inflammatory myofibroblastic tumors (IMT) [2, 3]. These chromosomal translocations induce the expression of various types of fusion proteins possessing the C‐terminal tyrosine kinase domain derived from the *ALK* gene. A variety of N‐terminal portions in ALK‐fusion proteins are caused by the pattern of chromosomal translocation. In the majority of ALK‐positive ALCL, a chromosomal disorder, such as t(2;5)(p23;q35), induces the expression of the nucleophosmin (NPM)‐ALK‐fusion protein [4]. Another chromosomal disorder, t(1;2)(q25;p23) has been reported in cases of IMT and a minor population of ALK‐positive ALCL, and a different fusion protein, tropomyosin 3 (TPM3)*‐*ALK is expressed [5, 6]. The expression of EML4‐ALK is caused by the chromosomal inversion in chromosome 2, inv (2) (p21; p23) in NSCLC [7]. Although these chromosomal abnormalities induce the expression of various types of ALK‐fusion proteins, their N‐terminal portions exhibit the same characteristic of causing the dimerization/oligomerization of ALK‐fusion proteins without any mitogenic stimulation, which results in the constitutive activation of ALK‐fusion proteins and their signaling cascades, for example, NPM‐ALK and its downstream molecules, such as STAT3, PI3K/Akt, and ERK [8, 9, 10, 11].

The subcellular localization of ALK‐fusion proteins is identified by the characteristics of their N‐terminal fused partners. NPM‐ALK and EML4‐ALK variant 3 are localized to both the cytoplasm and nucleus, and EML4‐ALK variant 1 and TPM3‐ALK are predominantly localized to the cytoplasm [8, 12, 13, 14]. Ceccon *et al*. [15] reported that NPM‐ALK was distributed in equal amounts between the cytoplasm and nucleus in ALCL cell lines and showed that only the cytoplasmic NPM‐ALK was catalytically active in these cells. On the other hand, we previously reported that the nuclear localization of NPM‐ALK was regulated by its kinase activity [16], suggesting that nuclear NPM‐ALK induces oncogenic features. Although NPM is a nucleolar protein possessing a nucleolar localization signal (NoLS), the N‐terminal portion of NPM‐ALK derived from the *NPM* gene lacks NoLS. Mason and colleagues demonstrated that NPM‐ALK localized not only to the cytoplasm, but also to the nucleolus of cultured cells derived from ALCL in the 1980s [12]; however, the precise function of nuclear NPM‐ALK has not yet been elucidated.

The nucleolus is a unique apparatus for rRNA transcription, modifications, and processing for the maturation of rRNA [17]. In the nucleolus, preribosomal RNA is transcribed from ribosomal DNA and processed appropriately to mature 18S, 5.8S, and 28S rRNA, which are assembled into the 40S or 60S ribosomal subunit. Furthermore, the function of the nucleolus varies because 70% of nucleolar proteins have a function that is unrelated to the production of ribosome subunits [17]. The nucleolus is also known to function as an apparatus for various tumor‐ and antitumor‐related events. Previous studies reported that the proto‐oncoprotein, c‐Myc localized to the nucleolus activated RNA polymerase I, suggesting that the acceleration of ribosome biogenesis affects oncogenicity [18, 19]. On the other hand, the ATM/ATR‐mediated activation of the tumor suppressor p53 was shown to occur in a nucleolus exposed to nucleolar stresses, such as a treatment with CX‐5461, an inhibitor of RNA polymerase I [20], suggesting that the nucleolar stresses caused by the disruption of ribosome biogenesis may be targets for cancer therapy [21].

In the present study, we observed that NPM‐ALK localized to the nucleolus in a manner that was dependent on its kinase activity through interactions with nucleophosmin 1 (NPM1). To reveal the function of NPM‐ALK in the nucleolus, we searched for the binding proteins of NPM‐ALK in the nucleolus and identified Epstein–Barr virus nuclear antigen 1‐binding protein 2 (EBP2), which is involved in rRNA biogenesis, and a nucleolar RNA helicase DDX21 as novel interactors of NPM‐ALK. Furthermore, we showed that EBP2 contributes to the cellular transformation provoked by NPM‐ALK through the suppression of p53 activation in the nucleolus of NPM‐ALK‐positive cells.

## 2 Materials and methods

### 2.1 Reagents

Alectinib and LY294002 were purchased from LC Laboratories (Woburn, MA, USA) and Merck Ltd. (Darmstadt, Germany), respectively. Hydrogen peroxide, methotrexate, and catalase were purchased from Nacalai Tesque (Kyoto, Japan). GDC‐0068 and rapamycin were purchased from Cayman Chemical (Ann Arbor, MI, USA) and Toronto Research Chemicals Inc. (Toronto, Canada), respectively. Anti‐β‐actin, anti‐Lamin B, anti‐Akt, anti‐p21, and anti‐p53 antibodies and sodium orthovanadate were purchased from Santa Cruz Biotechnology Inc. (Santa Cruz, CA, USA). To detect murine p53, an anti‐p53 antibody was purchased from Merck Millipore (Darmstadt, Germany). An anti‐NPM1 antibody and anti‐DDX21 antibody were obtained from Novus Biologicals (Centennial, CO, USA). An anti‐RPS7 antibody and anti‐RPL23 antibody were purchased from Abgent (San Diego, CA, USA). An anti‐Flag (M2) antibody, anti‐Fibrillarin antibody, and anti‐EBP2 antibody were purchased from were purchased from Sigma‐Aldrich (St. Louis, MO, USA), Abcam (Cambridge, MA, USA), and ProteinTech (Chicago, IL. USA), respectively. An anti‐RPL5 antibody and anti‐RPL11 antibody were purchased from Bethyl Laboratories (Montgomery, TX, USA). Other antibodies were purchased from Cell Signaling Technology (Danvers, MA, USA).

### 2.2 Plasmids

NPM1 cDNA and the cDNA encoding N‐terminal Flag NPM1 inserted into MSCV‐Puro retroviral vector. Retroviral vector for NPM‐ALK (K210R) was constructed using MSCV‐Puro‐N‐Flag NPM‐ALK as a template by mutagenesis PCR as previously described [16].

### 2.3 Cell culture, retrovirus infection, and transfection

The IL‐3‐dependent hematopoietic cell line Ba/F3 cells were cultured in RPMI‐1640 medium (Nacalai Tesque) containing 10% heat‐inactivated FBS (BioWest, Nuaille, France), 100 units·mL^−1^ penicillin (Nacalai Tesque), 100 μg·mL^−1^ streptomycin (Nacalai Tesque), 2 ng·mL^−1^ IL‐3 (PEPROTECH), and 5 μg·mL^−1^ puromycin (InVivoGen, San Diego, CA, USA). Ki‐JK cells and SUDH‐L1 cells, derived from NPM‐ALK‐positive ALCL patients, were cultured in RPMI‐1640 medium supplemented with 10% FBS, 100 units·mL^−1^ penicillin, and 100 μg·mL^−1^ streptomycin. HEK293T cells were cultured in Dulbecco's modified Eagle's medium (DMEM)–high glucose supplemented with 10% FBS, 100 units·mL^−1^ penicillin, and 100 μg·/mL^−1^ streptomycin. NPM1^−/−^/p53^−/−^ MEF and p53^−/−^ MEF were kindly gifted from Pier Paolo Pandolfi (Harvard University) and cultured in DMEM‐high glucose (Nacalai Tesque) supplemented with 10% FBS, 100 units·mL^−1^ penicillin, 100 μg·mL^−1^ streptomycin, and 55 μm 2‐mercaptoethanol (Nacalai Tesque). Ba/F3 cells were infected using RetroNectin (Takara Bio Inc., Shiga, Japan) and MEFs were infected using 10 μg·mL^−1^ polybrene as reported previously [16]. HEK293T cells were transfected with plasmids using polyethylenimine (PEI) Max (Polyscience inc. Warrington, PA, USA).

### 2.4 Fractionation of cells into cytosolic, nucleoplasmic, and nucleolar fractions

We conducted fractionation as described previously [22]. Briefly, cells were suspended in mild detergent buffer (20 mm Tris pH 7.4, 10 mm KCl, 3 mm MgCl_2_, 0.1% NP‐40, and 10% glycerol) and centrifuged at 1400 ***g*** at 4 °C for 10 min, and the resulting supernatant was prepared as the cytosolic fraction. Nuclear pellets were then resuspended in 0.25 m sucrose/10 mm MgCl_2_, layered over a cushion of 0.35 m sucrose/0.5 mm MgCl_2_, and centrifuged at 1400 ***g*** at 4 °C for 5 min. The resulting nuclear pellet was resuspended in 0.35 m sucrose/0.5 mm MgCl_2_ and sonicated on ice to disrupt nuclei and release nucleoli. The sonicate was layered over a cushion of 0.88 m sucrose/0.5 mm MgCl_2_ and then centrifuged at 2800 ***g*** at 4 °C for 10 min to obtain the nucleolar pellet. The resulting supernatant was retained as the nucleoplasmic fraction. Nucleoli were washed by resuspending in 0.5 mL of 0.35 m sucrose/0.5 mm MgCl_2_ and centrifuging at 2800 ***g*** at 4 °C for 5 min. The nucleolar pellet was resuspended in high salt RIPA buffer (50 mm Tris pH 7.4, 500 mm NaCl, 1% NP‐40, 0.5% deoxycholate) containing 16 units of DNase, incubated at 4 °C for 30 min, and then sonicated on ice. The sonicate was centrifuged at 20 000 ***g*** at 4 °C for 10 min, the supernatant was retained as the nucleolar extract, and the concentration of NaCl was adjusted to 150 mm by adding no salt RIPA buffer (50 mm Tris pH 7.4, 1% NP‐40, and 0.5% deoxycholate). The cytosolic, nucleoplasmic, and nucleolar fractions were denatured with Laemmli's sample buffer at 100 °C for 10 min.

### 2.5 Liquid chromatography–mass spectrometry (LC‐MS)

Purified protein complexes were precipitated by the addition of 20% trichloroacetic acid and resolved with 50 mm ammonium bicarbonate solution. To mask the thiol group, samples were treated with 10 mm DTT at 60 °C for 30 min and then reacted with 10 mm iodoacetamide. Protein digestion was performed by the addition of 1 U·mL^−1^ Trypsin Gold (Promega, Madison, WI, USA) overnight. Digested peptides were prepared by a treatment with de‐salting by Zip‐Tip (Merck Millipore, Burlington, MA, USA). Peptides were then separated by silica‐based reverse‐phase chromatography (gradient with acetonitrile: 0–60% in 0.1 m trifluoroacetic acid) and spotted on MTP AnchorChip 384 (Bruker Daltonics, Billerica, MA, USA) with mixing with α‐cyano‐4‐hydroxy cinnamic acid. Spotted peptides were analyzed by Autoflex speed (Bruker Daltonics), an analyzer for MALDI‐TOF/TOF with the protocol, Protein Scape (Bruker Daltonics), and proteins were identified by Mascot server (Matrix Science, Tokyo, Japan).

### 2.6 Immunoprecipitation and Immunoblot analysis

Cell lysates were prepared using Triton X‐100 lysis buffer (50 mm HEPES pH 7.5, 150 mm NaCl, and 1% Triton X‐100) supplemented with a protease inhibitor (Nacalai Tesque) and phosphatase inhibitor (Nacalai Tesque). For immunoprecipitation, cell lysates were incubated with anti‐DDDDK‐tag mAb‐Magnetic Beads (MBL, Nagoya, Japan) at 4 °C for 2 h, and beads were then washed with lysis buffer three times. Proteins were eluted in Laemmli's sample buffer by boiling. Regarding the immunoprecipitation of nucleolar lysates, nucleolar pellets were resuspended in high salt Triton X‐100 lysis buffer (50 mm HEPES pH 7.5, 500 mm NaCl, and 1% Triton X‐100) containing 16 units of DNase, incubated at 4 °C for 30 min, and then sonicated on ice. After centrifugation (15 000 r.p.m. at 4 °C for 10 min), the supernatant was retained as the nucleolar extract, and the concentration of NaCl was adjusted to 150 mm by adding no salt Triton X‐100 lysis buffer (50 mm HEPES pH 7.5, and 1% Triton X‐100). Nucleolar lysates were incubated with anti‐DDDDK‐tag mAb‐Magnetic Beads (MBL) at 4 °C for 4 h, and beads were then washed with lysis buffer three times. Bound proteins were eluted with 200 μg·mL^−1^ Flag peptide in Triton X‐100 lysis buffer and eluted fractions were denatured with Laemmli's sample buffer at 100 °C for 10 min for an immunoblot analysis. Regarding the immunoprecipitation of tyrosine‐phosphorylated proteins, cell lysates were prepared with Triton X‐100 lysis buffer supplemented with a protease inhibitor and phosphatase inhibitor. Cell lysates were homogenized using the ultrasonic homogenizer VP‐50 and centrifuged at 15 000 r.p.m. at 4 °C for 10 min to collect the supernatant. Protein G‐Magnetic beads (MBL) were precoated with an anti‐phospho‐tyrosine antibody according to the manufacturer's instructions. The supernatants were mixed with treated Protein G‐Magnetic beads at 4 °C for 4 h, and beads were then washed with lysis buffer three times. Proteins were eluted within Laemmli's sample buffer at 100 °C for 10 min.

SDS/PAGE, transfer, and immunoblotting were performed and the intensity of each band was quantified by imagej software [16].

### 2.7 Pervanadate treatment analysis

The stock solution of 100 mm Na_3_VO_4_ was prepared by dissolving Na_3_VO_4_ at pH 10 and boiling the solution until the yellow color had changed to a clear color. To prepare the 10 mm pervanadate solution, 100 mm Na_3_VO_4_ was diluted in PBS containing 0.1% H_2_O_2_ and incubated at 4 °C for 5 min. To remove excess H_2_O_2_, this solution was treated with catalase at a final concentration of 100 μg·mL^−1^ at room temperature for 5 min. The pervanadate solution was used within 5 min, and cells were incubated with 0.5 mm pervanadate solution for 30 min.

### 2.8 Water‐soluble tetrazolium assay and cell cycle analysis

Transduced Ba/F3 cells (5 × 10^4^ cells/100 μL) and Ki‐JK cells (2.5 × 10^4^ cells/100 μL) were cultured in a 96‐well plate for 24 and 48 h, respectively. After 10 μL Cell Count Reagent SF, Nacalai Tesque) was added, water‐soluble tetrazolium (WST) assay was performed as described previously [23]. For cell cycle analysis, after fixed with 70% (v/v) ethanol, cells were treated with RNase A (Nacalai Tesque) and added with propidium iodide (PI; Wako Pure Chemical Industries, Tokyo, Japan), and cell cycle analysis was performed as described previously [23].

### 2.9 RNA isolation and RT‐PCR (reverse transcriptase‐polymerase chain reaction)

Extraction of RNA, reverse transcriptase (RT), and quantitative real‐time PCR was performed as described previously [24]. PCR primer sequences were as follows: mouse p21, 5′‐GCAAAGTGTGCCGTTGTCTC‐3′ (forward) and 5′‐CGTCTCCGTGACGAAGTCAA‐3′ (reverse); mouse Rpl13a, 5′‐CCGGAAGCGGATGAATACCA‐3′ (forward) and 5′‐GAGGGATCCCATCCAACACC‐3′ (reverse); human p21, 5′‐TCTTGTACCCTTGTGCCTCG‐3′ (forward) and 5′‐ATCTGTCATGCTGGTCTGCC‐3′ (reverse); human Rpl13a, 5′‐CACGAGGTTGGCTGGAAGTA‐3′ (forward) and 5′‐CCGTAGCCTCATGAGCTGTT‐3′ (reverse).

#### 2.9.1 siRNA transfection

Transduced Ba/F3 cells and Ki‐JK cells were transfected with siRNA by electroporation using the Neon transfection system (WAKENYAKU Co., Ltd., Kyoto, Japan) according to the manufacturer's instructions. Briefly, 4 × 10^6^ transduced Ba/F3 cells and 3 × 10^6^ Ki‐JK cells were resuspended in 100 μL of R buffer containing 500 nm siRNA and then pulsed (1600 V, 20 ms, 1 pulse). After transfection, cells were immediately cultured in antibiotic‐free culture medium. Scrambled siRNA (Invitrogen, Carlsbad, CA, USA) was used as a negative control, and Stealth siRNAs targeting mouse EBP2, mouse Raptor, and human EBP2 were purchased from Invitrogen.

### 2.10 Metabolic labeling of nascent RNA

Transduced Ba/F3 cells were cultured for 4 h in growth medium containing 7.5 μCi ^3^H‐uridine (PerkinElmer, Waltham, MA, USA). After RNA isolation using Sepazol (Nacalai Tesque), total RNA (10 μg) was separated on a denaturing 1% agarose gel in MOPS buffer (20 mm MOPS pH 7.0, 5 mm sodium acetate, 1 mm EDTA). In order to partially undergo hydrolysis, the agarose gel was treated with 50 mm NaOH for 25 min, followed by neutralization using 200 mm sodium acetate. Separated RNA was transferred to the Hybond‐N^+^ nylon membrane (GE Healthcare, Waukesha, WI, USA) in the presence of 20 × SSC (3 m NaCl and 0.3 m sodium citrate, pH 7.3), and labeled RNA was visualized by fluorography using 2 m sodium salicylate [25]. The same Hybond‐N^+^ nylon membrane was stained using methylene blue.

### 2.11 Statistical analysis

Data were analyzed using spss Statistics software (version 23 for Macintosh, IBM Inc., Armonk, NY, USA) and presented as averages ± standard deviation (SD). *P* < 0.05 was considered to indicate a statistically significant difference.

## 3 Results

### 3.1 Kinase activity is essential for the nucleolar localization of NPM‐ALK

**Fig. 1:**
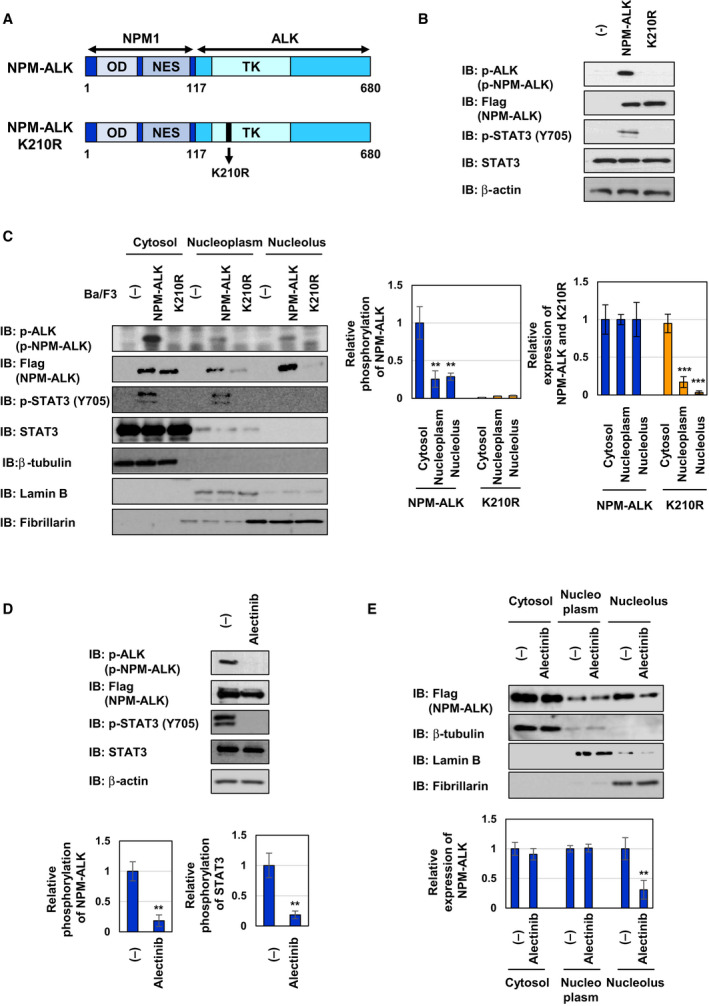
The kinase activity of NPM‐ALK is required for its nucleolar localization. (A) Structure of NPM‐ALK and its kinase dead mutant K210R. (B, C) Ba/F3 cells were infected with an empty virus (−) and expressed NPM‐ALK and its kinase dead mutant K210R by retroviral infection. These cells were named as control Ba/F3 cells (−), Ba/F3‐NPM‐ALK, and Ba/F3‐K210R, respectively. Whole‐cell lysates (B) and cytosolic, nucleoplasmic, and nucleolar fractions (C) were prepared. (D, E) Ba/F3‐NPM‐ALK were treated with alectinib (0.25 μm) for 4 h. Whole‐cell lysates (D) and cytosolic, nucleoplasmic, and nucleolar fractions (E) were prepared. (B–E) Immunoblotting was performed using an anti‐phospho‐ALK (Tyr1604), anti‐Flag, anti‐phospho‐STAT3 (Tyr705), anti‐STAT3, anti‐β‐actin, anti‐β‐tubulin, anti‐lamin B, or anti‐fibrillarin antibody. The relative phosphorylation or expression levels of NPM‐ALK and STAT3 are shown in the graphs. Results represent the mean ± SD of three independent experiments. **P < 0.01, ***P < 0.001 significantly different from the control group.

To investigate the subcellular localization of NPM‐ALK, we prepared two types of Ba/F3 cells expressing NPM‐ALK and its kinase dead mutant in which lysine at 210 in the ATP‐binding site was substituted to arginine (K210R) by retroviral infection (Fig. 1A). We named these cells ‘Ba/F3‐NPM‐ALK’ and ‘Ba/F3‐K210R’, respectively. As shown in Fig. 1B, NPM‐ALK was phosphorylated, whereas the K210R mutant was not, suggesting that the phosphorylation of NPM‐ALK was due to its auto‐phosphorylation. While the enforced expression of NPM‐ALK induced the phosphorylation of the downstream molecule, STAT3, the enforced expression of K210R failed to induce STAT3 phosphorylation in Ba/F3 cells (Fig. 1B). To identify the subcellular localization of NPM‐ALK and its K210R mutant, we prepared cell lysates under hypotonic conditions and separated them into cytosolic and nuclear fractions by centrifugation. NPM‐ALK was previously reported to be localized in the nucleolus [15]; therefore, we fractionated the nuclear fraction into the nucleoplasmic and nucleolar fractions. To evaluate fractionation, we analyzed the expression of β‐tubulin, lamin B, and fibrillarin as marker proteins for the cytosolic, nucleoplasmic, and nucleolar fractions, respectively (Fig. 1C). STAT3 in both the cytosol and nucleoplasm was phosphorylated by the enforced expression of NPM‐ALK, but not the K210R mutant. NPM‐ALK was detected in the cytosol, nucleoplasm, and nucleolus in Ba/F3 cells. In comparison with the phosphorylation level of NPM‐ALK in cytosol, the phosphorylation level of NPM‐ALK in nucleoplasm and nucleolus was significantly decreased. On the other hand, the K210R mutant was predominantly localized in the cytosol and slightly localized in the nucleoplasm, while the nucleolar localization of K210R mutant was negligible. These results suggest that the nucleolar localization of NPM‐ALK depends on its kinase activity but the catalytic activity of nucleolar NPM‐ALK is lower than that of cytoplasmic NPM‐ALK (Fig. 1C). A treatment with the ALK inhibitor, alectinib prohibited the phosphorylation of NPM‐ALK and STAT3 and markedly diminished the nucleolar localization of NPM‐ALK, which was consistent with the results obtained on the localization of NPM‐ALK and the K210R mutant (Fig. 1D,E). These results indicate that the kinase activity of NPM‐ALK is essential for its nucleolar localization.

### 3.2 The interaction with NPM1 is essential for the nuclear localization of NPM‐ALK

**Fig. 2:**
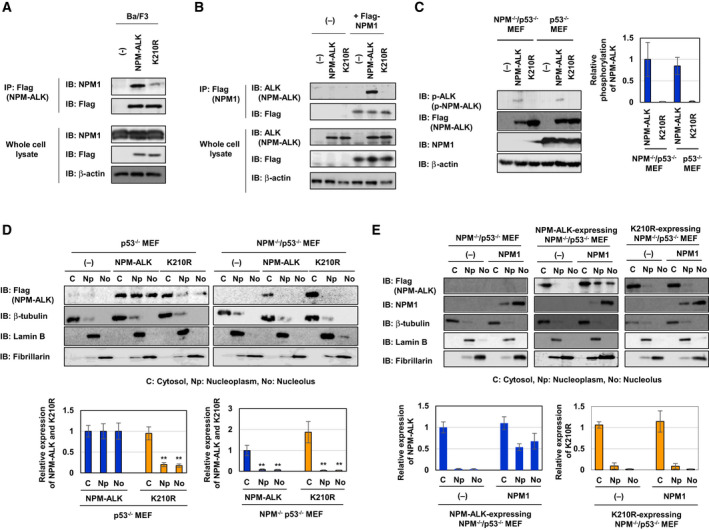
NPM1 is required for the nuclear localization of NPM‐ALK. (A) Cell lysates were prepared from control Ba/F3 cells (−), Ba/F3‐NPM‐ALK, and Ba/F3‐K210R and were then immunoprecipitated with an anti‐Flag antibody. Immunoprecipitates and whole‐cell lysates were immunoblotted with an anti‐NPM1, anti‐Flag, or anti‐β‐actin antibody. (B) HEK293T cells were transiently transfected with an empty vector (−) or plasmids bearing NPM‐ALK, its kinase dead mutant K210R or Flag‐tagged NPM1. Cell lysates were immunoprecipitated with an anti‐Flag antibody. Immunoprecipitates and whole‐cell lysates were immunoblotted with an anti‐ALK, anti‐Flag, or anti‐β‐actin antibody. (C, D) NPM1^−/−^/p53^−/−^MEF and p53^−/−^MEF were infected with an empty virus (−) and expressed NPM‐ALK and its kinase dead mutant K210R by retroviral infection. (C) Whole‐cell lysates were prepared and immunoblotted with an anti‐phospho‐ALK (Tyr1604), anti‐Flag, anti‐NPM1, or anti‐β‐actin antibody. The relative phosphorylation levels of NPM‐ALK and its kinase dead mutant K210R are shown in the graph. Results represent the mean ± SD of three independent experiments. (D) Cytosolic, nucleoplasmic, and nucleolar fractions of transduced NPM1^−/−^/p53^−/−^MEF and p53^−/−^MEF were prepared and immunoblotted with an anti‐Flag, anti‐β‐tubulin, anti‐Lamin B, or anti‐Fibrillarin antibody. C, Np, and No indicate the cytosolic fraction, nucleoplasmic fraction, and nucleolar fraction, respectively. The relative expression levels of NPM‐ALK and its kinase dead mutant K210R are shown in the graphs. Results represent the mean ± SD of three independent experiments. **P < 0.01 significantly different from the control group. (E) NPM1^−/−^/p53^−/−^MEF and NPM1^−/−^/p53^−/−^MEF expressing NPM‐ALK and NPM1^−/−^/p53^−/−^MEF expressing the kinase dead mutant of NPM‐ALK K210R were infected with an empty virus (−) and expressed NPM1 by retroviral infection. Cytosolic, nucleoplasmic, and nucleolar fractions from transduced MEF were prepared and immunoblotted with an anti‐Flag, anti‐NPM1, anti‐β‐tubulin, anti‐Lamin B, or anti‐Fibrillarin antibody. C, Np, and No indicate the cytosolic, nucleoplasmic, and nucleolar fractions, respectively. The relative expression levels of NPM‐ALK and its kinase dead mutant K210R are shown in the graphs. Results represent the mean ± SD of three independent experiments.** indicates *P* < 0.01.

NPM1, which contains the oligomerization domain, nuclear export signal, nuclear localization signal, and NoLS, mainly localizes to the nucleolus and shuttles between the cytosol and nucleus [26]. Since NPM‐ALK contains the oligomerization domain, as shown in Fig. 1A, we hypothesized that it localized to the nucleus and nucleolus by interacting with NPM1. To test this hypothesis, we examined the interaction between NPM‐ALK and NPM1 using a co‐immunoprecipitation assay. NPM‐ALK more strongly interacted with NPM1 than the K210R mutant (Fig. 2A). To confirm this interaction, HEK293T cells were expressed Flag‐tagged NPM1 and NPM‐ALK or K210R mutant and immunoprecipitation was performed using anti‐Flag antibody. Flag‐tagged NPM1 interacted with NPM‐ALK but not K210R mutant (Fig. 2B), suggesting that the kinase activity of NPM‐ALK facilitates the interaction with NPM1. We then investigated whether NPM1 regulates the localization of NPM‐ALK by using NPM1‐deficient cells. A previous study reported that NPM^−/−^ MEFs do not proliferate and exhibit a senescence‐like phenotype in a p53‐dependent manner [27]; therefore, we utilized NPM^−/−^/p53^−/−^ MEFs and p53^−/−^ MEFs in which NPM‐ALK and the K210R mutant were forcibly expressed (Fig. 2C). Using the same procedure, we examined the subcellular localization of NPM‐ALK and its K210R mutant in these MEFs. While NPM‐ALK localized to the cytosol, nucleoplasm, and nucleolus in p53^−/−^ MEFs, the K210R mutant predominantly localized to the cytosol and slightly to the nucleoplasm and nucleolus in p53^−/−^ MEFs, confirming that the kinase activity of NPM‐ALK was required for the nuclear localization of NPM‐ALK (Fig. 2D, left). On the other hand, in NPM^−/−^/p53^−/−^ MEFs, NPM‐ALK failed to localize to the nucleoplasm and nucleolus, suggesting the requirement of NPM1 for the nuclear and nucleolar localization of NPM‐ALK (Fig. 2D, right). Furthermore, the enforced expression of NPM1 in a NPM1‐deficient background effectively restored the nuclear and nucleolar localization of NPM‐ALK, but not K210R (Fig. 2E). On the other hand, the phosphorylation of NPM‐ALK and STAT3 was not altered in the absence or presence of NPM1 (Fig. S1). In addition, the present results confirmed that K210R was not translocated into the nucleus or nucleolus under any conditions, such as the presence or absence of NPM1 (Fig. 2E). Therefore, these results suggest that NPM1 reacts only with activated NPM‐ALK and induces the localization of activated NPM‐ALK to the nucleoplasm and nucleolus.

### 3.3 NPM‐ALK interacts with EBP2 and DDX21 in the nucleolus and the phosphorylation of EBP2 and DDX21 was enhanced by the enforced expression of NPM‐ALK

**Fig. 3:**
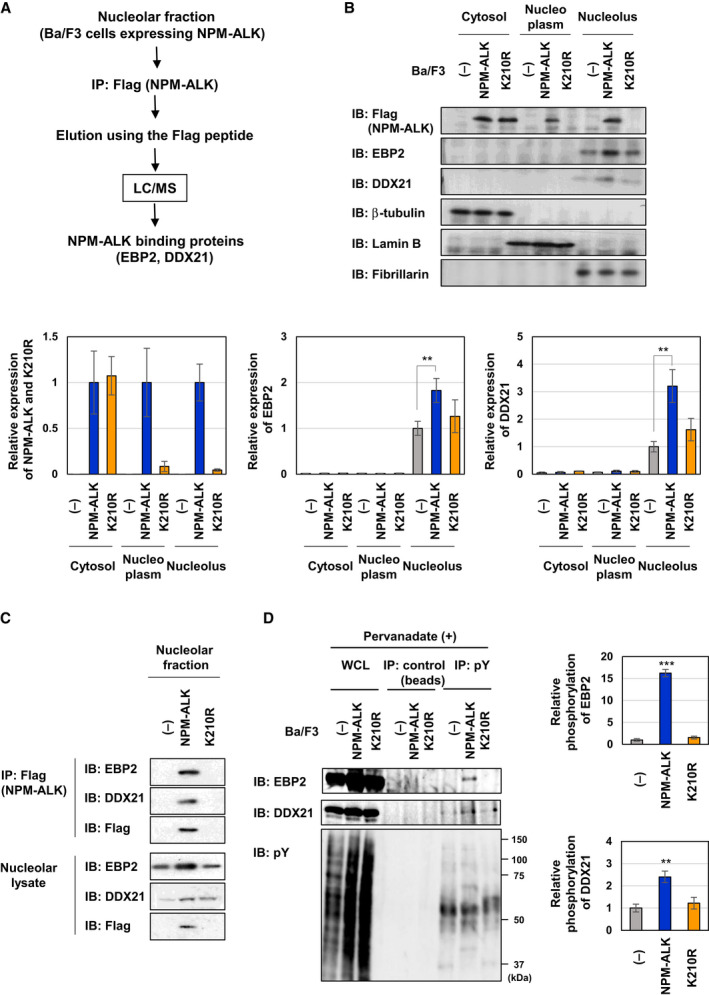
NPM‐ALK interacts with EBP2 in the nucleolus and induces tyrosine phosphorylation. (A) Scheme of the experimental procedure for the identification of nucleolar NPM‐ALK binding partners. (B) Cytosolic, nucleoplasmic, and nucleolar fractions were prepared from control Ba/F3 cells (−), Ba/F3‐NPM‐ALK, and Ba/F3‐K210R. Each fraction was analyzed by immunoblotting with an anti‐Flag, anti‐DDX21, anti‐EBP2, anti‐β‐tubulin, anti‐Lamin B, or anti‐Fibrillarin antibody. The relative expression levels of NPM‐ALK, its kinase dead mutant K210R, EBP2, and DDX21 are shown in the graphs. Results represent the mean ± SD of three independent experiments. ** indicates *P* < 0.01. (C) Nucleolar fractions were prepared from control Ba/F3 cells (−), Ba/F3‐NPM‐ALK, and Ba/F3‐K210R. Nucleolar fractions were immunoprecipitated with an anti‐Flag antibody and immunoprecipitates were eluted with lysis buffer containing the Flag peptide. Eluted samples and nucleolar lysates were immunoblotted with an anti‐DDX21, anti‐EBP2, or anti‐Flag antibody. (D) Control Ba/F3 cells (−), Ba/F3‐NPM‐ALK, and Ba/F3‐K210R were treated with 0.5 mmpervanadate for 30 min. Cell lysates were prepared and immunoprecipitated with control beads or an anti‐phospho‐tyrosine (pY) antibody. Immunoprecipitates were immunoblotted with an anti‐EBP2, anti‐DDX21, or anti‐phospho‐tyrosine (pY) antibody. The relative phosphorylation levels of EBP2 and DDX21 are shown in the graphs. Results represent the mean ± SD of three independent experiments. **P < 0.01, ***P < 0.001 significantly different from the control group of Ba/F3 cells.

As shown in Fig. 2, NPM‐ALK localized to the nucleus and nucleolus in a manner that was dependent on its kinase activity, suggesting the importance of nucleolar NPM‐ALK for tumorigenesis. To clarify the function of nucleolar NPM‐ALK, we aimed to identify NPM‐ALK binding molecules in the nucleolus. The nucleolus was isolated from control Ba/F3 and Ba/F3‐NPM‐ALK, nucleolar lysates were utilized for immunoprecipitation with the anti‐Flag antibody, and protein complexes were then eluted by the Flag peptide. By using mass spectrometry, we identified two proteins binding to NPM‐ALK, EBP2 and nucleolar RNA helicase DDX21 (Fig. 3A). As shown in Fig. 3B, DDX21 and EBP2 only localized to the nucleolus. We then investigated whether DDX21 and EBP2 interact with NPM‐ALK using nucleolar fractions by co‐immunoprecipitation and clearly detected the interaction of DDX21 and EBP2 with NPM‐ALK, but not the K210R mutant in the nucleolus (Fid. 3C). The significant tyrosine phosphorylation of EBP2 was detected in Ba/F3‐NPM‐ALK but not in Ba/F3‐K210R under the treatment with pervanadate, a protein–tyrosine phosphatase inhibitor, leading to increases in intracellular tyrosine phosphorylation (Fig. S2, Fig. 3D). The tyrosine phosphorylation of DDX21 was also slightly increased by the enforced expression of NPM‐ALK, but not the K210R mutant (Fig. 3D). These results suggested that NPM‐ALK binds to EBP2 and DDX21 in the nucleolus, and caused their tyrosine phosphorylation in the kinase activity of NPM‐ALK‐dependent manner.

### 3.4 EBP2 is partially involved in NPM‐ALK‐induced 28S rRNA biogenesis

**Fig. 4:**
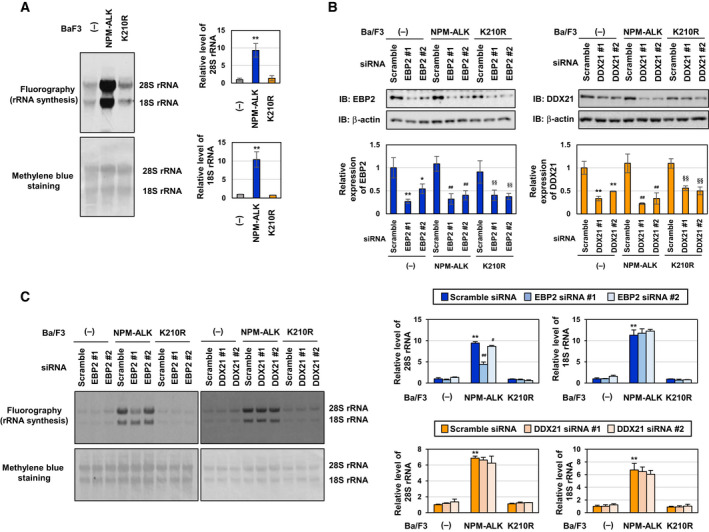
Knockdown of EBP2 inhibits 28S rRNA biogenesis in Ba/F3 cells expressing NPM‐ALK. (A) Control Ba/F3 cells (−), Ba/F3‐NPM‐ALK, and Ba/F3‐K210R cells were metabolically labeled with [^3^H]‐uridine for 4 h. RNA was isolated and analyzed by agarose gel electrophoresis and fluorography (upper). Methylene blue staining was performed (bottom). (B, C) Control Ba/F3 cells (−), Ba/F3‐NPM‐ALK, and Ba/F3‐K210R were transfected with control scrambled siRNA and two kinds of siRNA targeting EBP2 (EBP2 siRNA #1, or EBP2 siRNA #2) and then incubated for 20 h. (B) Whole‐cell lysates were prepared and immunoblotted with an anti‐EBP2 antibody or anti‐β‐actin antibody. The relative expression levels of EBP2 and DDX21 are shown in the graphs. Results represent the mean ± SD of three independent experiments. *P < 0.05, **P < 0.001 significantly different from the control group of Ba/F3 cells transfected with scrambled siRNA.^##^ *P* < 0.01 significantly different from the group of Ba/F3‐ NPM‐ALK transfected with scrambled siRNA.^§§^ *P* < 0.01 significantly different from the group of Ba/F3‐K210R transfected with scrambled siRNA. (C) Cells were metabolically labeled with [^3^H]‐uridine for 4 h. Total RNA was isolated and analyzed by agarose gel electrophoresis and fluorography (upper). Methylene blue staining was performed (bottom). Quantification data of synthesized 28S rRNA and 18S rRNA were shown in the graph, with values from control Ba/F3 cells transfected with scrambled siRNA being set to 1 (*n* = 3). **P < 0.01 significantly different from the group of control Ba/F3 cells (−) transfected with scrambled siRNA.^##^ *P* < 0.01 significantly different from the group of Ba/F3‐NPM‐ALK transfected with scrambled siRNA.

Ribosomal biogenesis generally occurs in the nucleolus. In yeast, EBP2 is involved in preribosomal RNA processing, ribosomal subunit assembly, and cell growth [28]. We investigated the effects of NPM‐ALK and EBP2 and DDX21 on rRNA biogenesis. Control Ba/F3 cells (−), Ba/F3‐NPM‐ALK, and Ba/F3‐K210R were metabolically labeled with ^3^H‐uridine, and extracted total RNA was then separated by agarose gel electrophoresis and visualized using fluorography. While the enforced expression of NPM‐ALK accelerated the biogenesis of 28S rRNA and 18S rRNA, the enforced expression of the K210R mutant failed to induce rRNA biogenesis, suggesting that the kinase activity of NPM‐ALK is critical for rRNA biogenesis (Fig. 4A). We then investigated the effects of EBP2 and DDX21 on NPM‐ALK‐accelerated rRNA biogenesis using siRNA targeting EBP2 (Fig. 4B). The knockdown of EBP2 using siRNAs, EBP2#1, and EBP2#2 led to an approximately 25% and 10% reduction, respectively, in 28S rRNA biogenesis in Ba/F3‐NPM‐ALK. Alternatively, the knockdown of EBP2 had no effect on NPM‐ALK‐induced 18S rRNA biogenesis. In addition, the knockdown of DDX21 had no effect on biogenesis of 28S rRNA and 18S rRNA in Ba/F3‐NPM‐ALK (Fig. 4C). These results indicate that EBP2 is partially, but specifically involved in NPM‐ALK‐induced 28S rRNA biogenesis. Previous studies reported that impairments in ribosomal biogenesis, including the inhibition of pre‐rRNA transcription and rRNA processing, resulted in the cytosolic distribution of ribosomal proteins from nucleolus, such as RPL5, L11, L23, and S7 [29, 30, 31, 32, 33]. However, the knockdown of EBP2 had no effect on the expression levels of RPL5, RPL11, RPL23, or RPS7 in the nucleolus or nucleoplasm in Ba/F3‐NPM‐ALK (Fig. S3).

### 3.5 EBP2 is required for NPM‐ALK‐induced cell proliferation through the inactivation of p53

**Fig. 5:**
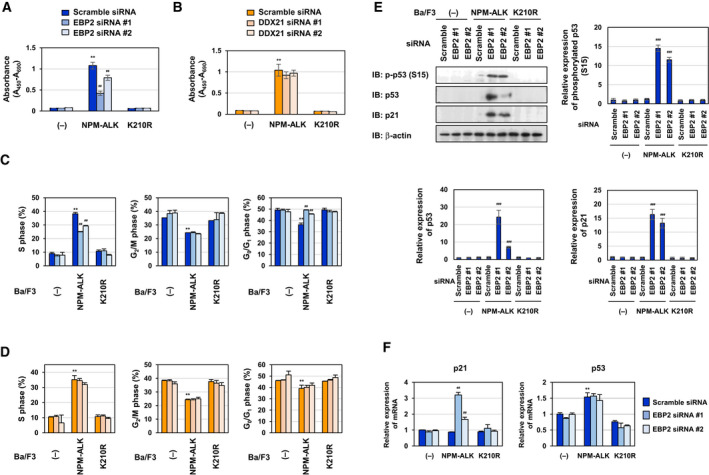
Knockdown of EBP2 induces G_0_/G_1_‐phase cell cycle arrest through p53 activation in Ba/F3 cells expressing NPM‐ALK. Control Ba/F3 cells (−), Ba/F3‐NPM‐ALK, and Ba/F3‐K210R were transfected with control scrambled siRNA, two kinds of siRNA targeting EBP2 (EBP2 siRNA #1 or EBP2 siRNA #2) and two kinds of siRNA targeting DDX21 (DDX21 siRNA #1 or DDX21 siRNA #2), and were then incubated for 20 h. (A, B) Cells (5 × 10^4^ cells/100 μL) were cultured for 24 h and the proliferation rate was measured using the WST‐1 assay (*n* = 4). Error bars represent the SD of the mean. **P < 0.01 significantly different from the group of control Ba/F3 cells transfected with scrambled siRNA.^##^ *P* < 0.01 significantly different from the group of Ba/F3‐NPM‐ALK transfected with scrambled siRNA. (C, D) Cells were fixed, treated with propidium iodide, and subjected to a flow cytometric analysis. The ratios of cells in the S phase, G_0_/G_1_phase, and G_2_/M phase were graphed. Data were expressed as means ± SD (*n* = 3). **P < 0.01 significantly different from the group of control Ba/F3 cells transfected with scrambled siRNA.^##^ *P* < 0.01 significantly different from the group of Ba/F3‐NPM‐ALK transfected with scrambled siRNA. (E) Whole‐cell lysates were immunoblotted with an anti‐phospho‐p53 (Ser15), anti‐p53, anti‐p21, anti‐EBP2, or anti‐β‐actin antibody. The relative phosphorylation or expression levels of p53 and p21 are shown in the graphs. Results represent the mean ± SD of three independent experiments.^###^ *P* < 0.001 significantly different from the control group of Ba/F3‐NPM‐ALK transfected with scrambled siRNA. (F) Total RNA was prepared and the expression of*p21*and*p53*mRNA was analyzed by quantitative real‐time PCR (*n* = 3).*Rpl13a*mRNA was analyzed as an internal control. Error bars represent the SD of the mean. **P < 0.01 significantly different from the group of control Ba/F3 cells transfected with scrambled siRNA.^##^ *P* < 0.01 significantly different from the group of Ba/F3‐NPM‐ALK transfected with scrambled siRNA.

In order to elucidate the functions of EBP2 and DDX21 in Ba/F3‐NPM‐ALK, we first looked into the effects of the knockdown of EBP2 and DDX21 on NPM‐ALK‐induced cell proliferation using the WST assay. The growth of Ba/F3‐NPM‐ALK was significantly suppressed by the knockdown of EBP2, but not by the knockdown of DDX21 (Fig. 5A,B). We then examined the effects of the knockdown of EBP2 and DDX21 on the cell cycle in control Ba/F3 cells (−), Ba/F3‐NPM‐ALK, and Ba/F3‐K210R. In comparisons with the cell cycle patterns of control cells (−), the ratio of the S phase was significantly higher and that of the G_2_/M phase and G_0_/G1 phase was markedly lower in Ba/F3‐NPM‐ALK. However, these alterations were not observed in Ba/F3‐K210R. The knockdown of EBP2 canceled these NPM‐ALK‐induced alterations in the ratio of S phase. Furthermore, the knockdown of EBP2 increased the population of the G_0_/G_1_ phase, suggesting that EBP2 is required for the entry and progression of the cell cycle from the G_0_/G_1_ phase to the S phase (Fig. 5C). The knockdown of EBP2 had no effect on cell cycle in control Ba/F3 cells (−) and Ba/F3‐K210R (Fig. 5C). Contrary, the knockdown of DDX21 did not affect cell cycle in control cells (−), Ba/F3‐NPM‐ALK, and Ba/F3‐K210R (Fig. 5D). Moreover, the knockdown of EBP2 induced the accumulation of p53, which was phosphorylated at Ser15, in Ba/F3‐NPM‐ALK, but not in control Ba/F3 cells (−) or Ba/F3‐K210R (Fig. 5E). The phosphorylation of p53 at Ser15 is a marker for the transcriptional activation of p53 [34], and this was supported by the results obtained on the expression of the p21 protein and *p21* mRNA (Fig. 5E,F). The expression level of p53 mRNA was increased in Ba/F3‐NPM‐ALK but not Ba/F3‐K210R in comparison with that in control Ba/F3 cells (−), but the knockdown of EBP2 had no effect on the expression of p53 mRNA in Ba/F3‐NPM‐ALK, suggesting that EBP2 knockdown‐induced p53 accumulation is not due to the activation of p53 promoter (Fig. 5F). Since the marker of DNA damage, γ‐H2AX was not detected in Ba/F3‐NPM‐ALK in which EBP2 was knocked down, the activation of p53 induced by the knockdown of EBP2 may not have been due to DNA damage in Ba/F3‐NPM‐ALK (Fig. S4).

### 3.6 EBP2 negatively regulates p53 activity by inhibiting NPM‐ALK‐induced activation of the Akt/mTORC1 signaling pathway

**Fig. 6:**
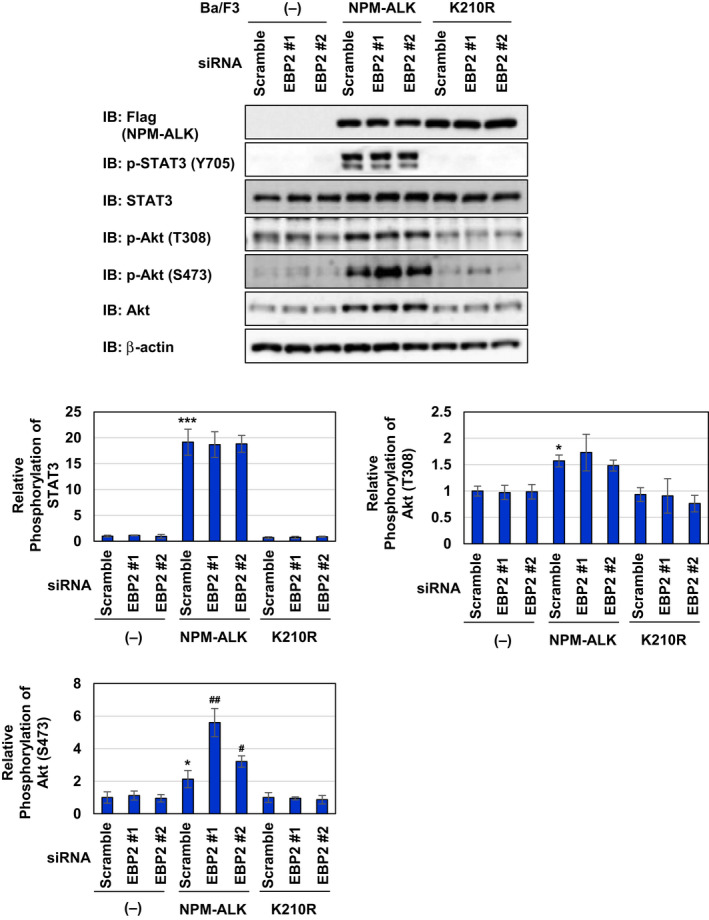
Knockdown of EBP2 enhances phosphorylation of Akt at S473 in Ba/F3 cells expressing NPM‐ALK. Control Ba/F3 cells (−), Ba/F3‐NPM‐ALK, and Ba/F3‐K210R were transfected with control scrambled siRNA and two kinds of siRNA targeting EBP2 (EBP2 siRNA #1 or EBP2 siRNA #2). Twenty hours after transfection, whole‐cell lysates were prepared and immunoblotted with an anti‐Flag, anti‐phospho‐STAT3 (Tyr705), anti‐STAT3, anti‐phospho‐Akt (Thr308), anti‐phospho‐Akt (Ser473), anti‐Akt, or anti‐β‐actin antibody. The relative phosphorylation levels of STAT3 and Akt are shown in the graphs. Results represent the mean ± SD of three independent experiments. *P < 0.05, ***P < 0.001 significantly different from the control group of Ba/F3 cells transfected with scrambled siRNA.^#^ *P* < 0.05,^##^ *P* < 0.01 significantly different from the group of Ba/F3‐NPM‐ALK transfected with scrambled siRNA.

**Fig. 7:**
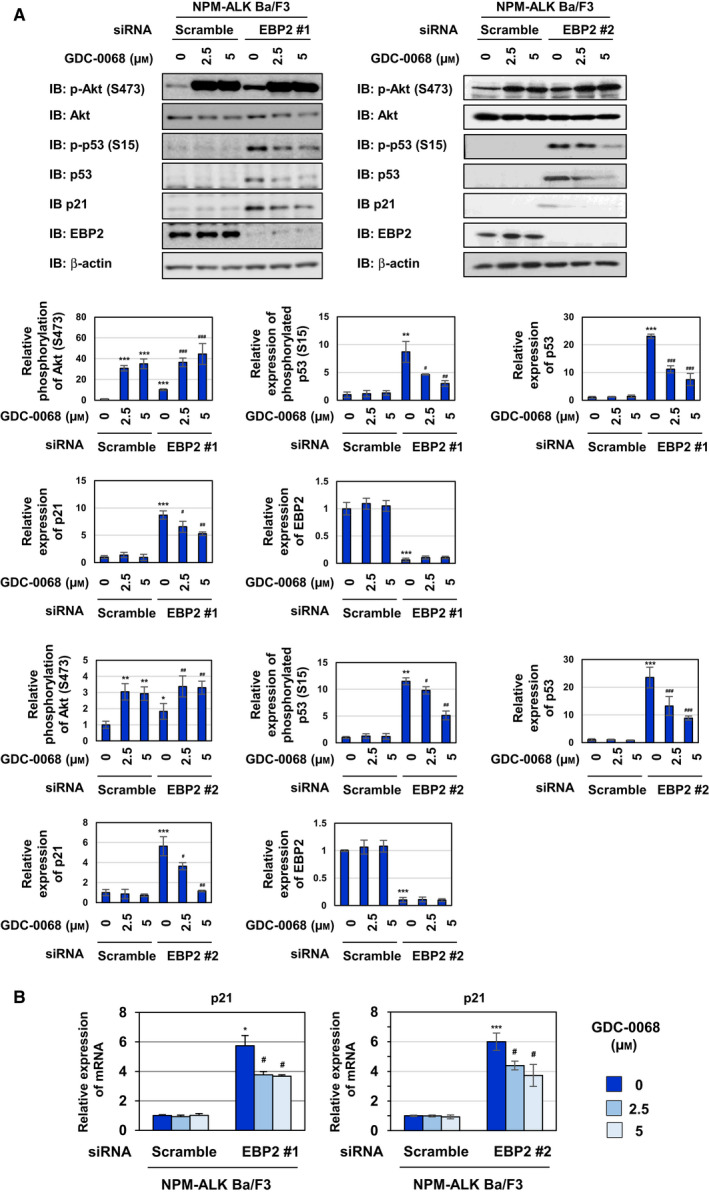
Knockdown of EBP2 activates p53 through Akt in Ba/F3 cells expressing NPM‐ALK. Ba/F3‐NPM‐ALK was transfected with control scrambled siRNA and two kinds of siRNA targeting EBP2 (EBP2 siRNA #1 or EBP2 siRNA #2). Fourteen hours after transfection, cells were treated with GDC‐0068 (2.5 and 5 μm) for 6 h. (A) Whole‐cell lysates were immunoblotted with an anti‐phospho‐Akt (Ser473), anti‐Akt, anti‐phospho‐p53 (Ser15), anti‐p53, anti‐p21, anti‐EBP2, or anti‐β‐actin antibody. The relative phosphorylation or expression levels of Akt, p53, p21, and EBP2 are shown in the graphs. Results represent the mean ± SD of three independent experiments. *P < 0.05, **P < 0.01, ***P < 0.001 significantly different from the control group of Ba/F3‐NPM‐ALK transfected with scrambled siRNA.^#^ *P* < 0.05,^##^ *P* < 0.01,^###^ *P* < 0.001 significantly different from the group of Ba/F3‐NPM‐ALK transfected with EBP2 siRNA #1 or EBP2 siRNA #2. (B) Total RNA was prepared and the expression of*p21*mRNA was analyzed by quantitative real‐time PCR (*n* = 3).*Rpl13a*mRNA was analyzed as an internal control. Error bars represent the SD of the mean. *P < 0.05; ***P < 0.001 significantly different from the group of Ba/F3‐NPM‐ALK transfected with scrambled siRNA.^#^ *P* < 0.05 significantly different from the group of Ba/F3‐NPM‐ALK transfected with EBP2 siRNA #1 or EBP2 siRNA #2.

In addition to STAT3, a downstream molecule, such as Akt, is regulated by NPM‐ALK. NPM‐ALK induced the activation of Akt, as evidenced by its phosphorylation at Thr308 and Ser473 in spite of the upregulated expression of the Akt protein. On the other hand, the K210R mutant failed to exert similar effects on Akt (Fig. 6). To elucidate the molecular mechanisms by which the knockdown of EBP2 induced p53 activation in Ba/F3‐NPM‐ALK, we investigated whether the knockdown of EBP2 affected the NPM‐ALK‐induced signaling pathway, such as the activation of STAT3 and PI3K/Akt. Although the knockdown of EBP2 did not affect the phosphorylation of STAT3 or Akt at Thr308 in Ba/F3‐NPM‐ALK, the NPM‐ALK‐induced phosphorylation of Akt at Ser473 was increased by the knockdown of EBP2 using siRNAs, EBP2#1, and EBP2#2 (Fig. 7A). The activity of Akt was previously shown to be mainly regulated by phosphorylation at Thr308 and Ser473, both of which are required for the maximal activation of Akt [35]. These results revealed that EBP2 negatively regulated Akt activation, and its knockdown induced the enhanced activation of Akt in Ba/F3‐NPM‐ALK. We then investigated the effects of the enhanced activation of Akt induced by the knockdown of EBP2 in Ba/F3‐ NPM‐ALK using GDC‐0068, which is an ATP‐competitive Akt inhibitor. After the transfection of siRNAs targeting EBP2, EBP2#1, and EBP2#2, we treated Ba/F3‐NPM‐ALK with GDC‐0068. Previous studies reported that GDC‐0068 protected Akt from phosphatases targeting phosphorylated Ser473 by stabilizing its conformation [36, 37], and the treatment with GDC‐0068 increased the phosphorylation of Akt at Ser473 in both Ba/F3‐NPM‐ALK transfected scramble siRNA and EBP2 siRNAs in the present study (Fig. 7A). p53 accumulation and p21 expression caused by the knockdown of EBP2 using siRNAs, EBP2#1, and EBP2#2 were markedly inhibited by GDC‐0068 in Ba/F3‐NPM‐ALK (Fig. 7A,B). These results suggest that EBP2 negatively regulates p53 activity by inhibiting the excessive activation of Akt induced by NPM‐ALK, leading to the marked proliferation of transformed cells by NPM‐ALK.

**Fig. 8:**
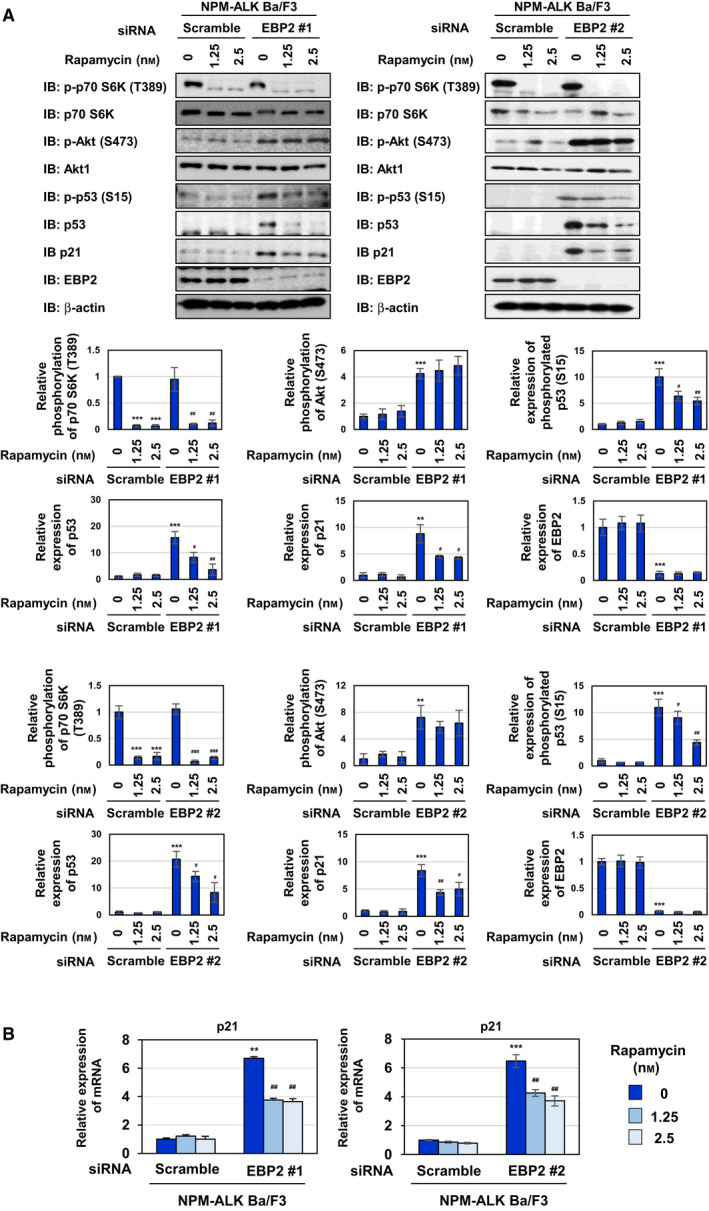
The EBP2 knockdown‐induced p53 activation is inhibited by Rapamycin in Ba/F3 cells expressing NPM‐ALK. Ba/F3‐NPM‐ALK was transfected with control scrambled siRNA and two kinds of siRNA targeting EBP2 (EBP2 siRNA #1 or EBP2 siRNA #2). Fourteen hours after transfection, cells were treated with rapamycin (1.25 and 2.5 nm) for 6 h. (A) Whole‐cell lysates were immunoblotted with an anti‐phospho‐p70 S6 kinase (Thr389), anti‐p70 S6 kinas, anti‐phospho‐Akt (Ser473), anti‐Akt, anti‐phospho‐p53 (Ser15), anti‐p53, anti‐p21, anti‐EBP2, or anti‐β‐actin antibody. The relative phosphorylation or expression levels of p70 S6K, Akt, p53, p21, and EBP2 are shown in the graphs. Results represent the mean ± SD of three independent experiments. **P < 0.01, ***P < 0.001 significantly different from the control group of Ba/F3‐NPM‐ALK transfected with scrambled siRNA.^#^ *P* < 0.05,^##^ *P* < 0.01,^###^ *P* < 0.01 significantly different from the group of Ba/F3‐NPM‐ALK transfected with EBP2 siRNA #1 or EBP2 siRNA #2. (B) Total RNA was prepared and the expression of*p21*mRNA was analyzed by quantitative real‐time PCR (*n* = 3).*Rpl13a*mRNA was analyzed as an internal control. Error bars represent the SD of the mean. **P < 0.01; ***P < 0.001 significantly different from the group of Ba/F3‐NPM‐ALK transfected with scrambled siRNA.^##^ *P* < 0.01 significantly different from the group of Ba/F3‐NPM‐ALK transfected with EBP2 siRNA #1 or EBP2 siRNA #2.

**Fig. 9:**
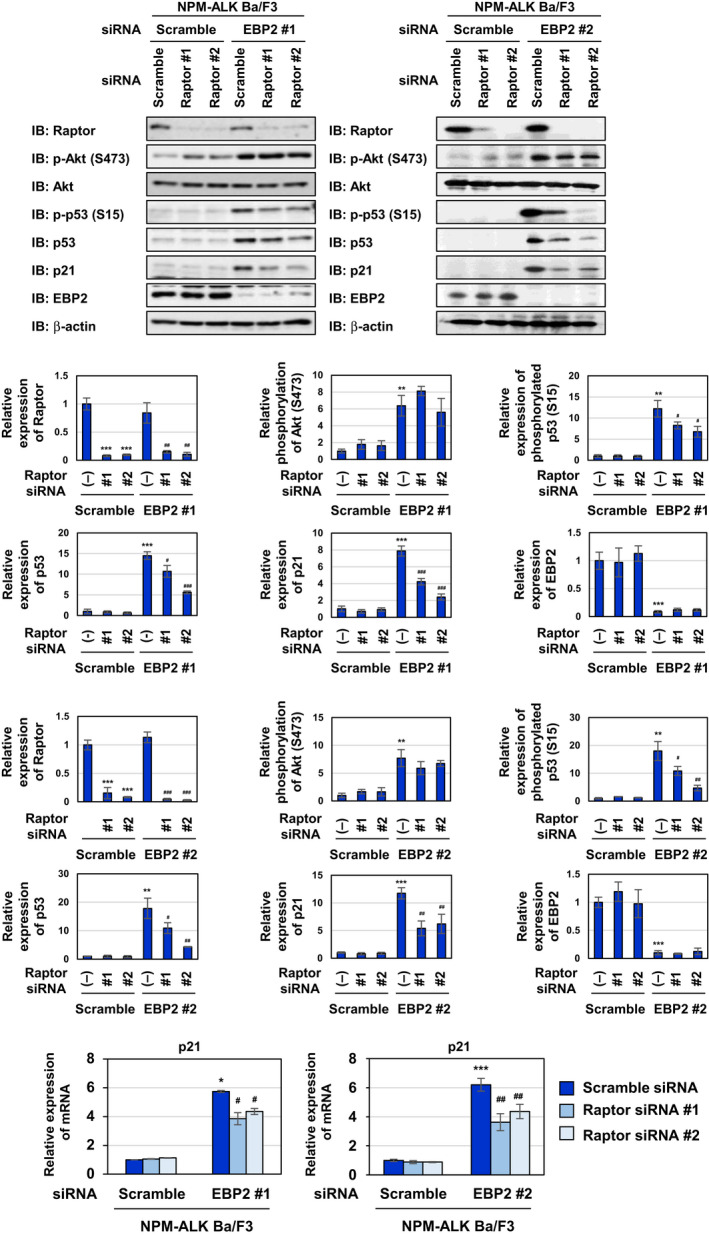
Knockdown of EBP2 activates p53 through the Akt‐mTORC1 pathway in Ba/F3 cells expressing NPM‐ALK. Ba/F3‐NPM‐ALK were transfected with scrambled siRNA and/or two kinds of siRNA targeting EBP2 (EBP2 siRNA #1 or EBP2 siRNA #2) and/or two kinds of siRNA targeting Raptor (Raptor siRNA #1 or Raptor siRNA #2), and then incubated for 20 h. (A) Whole‐cell lysates were immunoblotted with an anti‐Raptor, anti‐phospho‐Akt (Ser473), anti‐Akt, anti‐phospho‐p53 (Ser15), anti‐p53, anti‐p21, anti‐EBP2, or anti‐β‐actin antibody. The relative phosphorylation or expression levels of Raptor, Akt, p53, p21, and EBP2 are shown in the graphs. Results represent the mean ± SD of three independent experiments. **P < 0.01, ***P < 0.001 significantly different from the control group of Ba/F3‐NPM‐ALK transfected with scrambled siRNA.^#^ *P* < 0.05,^##^ *P* < 0.01,^###^ *P* < 0.001 significantly different from the group of Ba/F3‐NPM‐ALK transfected with EBP2 siRNA #1 or EBP2 siRNA #2. (B) Total RNA was prepared and the expression of*p21*mRNA was analyzed by quantitative real‐time PCR (*n* = 3).*Rpl13a*mRNA was analyzed as an internal control. Error bars represent the SD of the mean. *P < 0.05, ***P < 0.001 significantly different from the group of Ba/F3‐NPM‐ALK transfected with scrambled siRNA.^#^ *P* < 0.05,^##^ *P* < 0.01 significantly different from the group of Ba/F3‐ NPM‐ALK transfected with EBP2 siRNA #1 or EBP2 siRNA #2.

To gain further insights into the mechanisms by which EBP2 knockdown‐activated Akt causes p53 activation, we focused on the mechanistic target of rapamycin (mTOR) complex 1 (mTORC1), a target molecule of Akt [38, 39]. However, the knockdown of EBP2 using siRNAs, EBP2#1, and EBP2#2 failed to induce mTORC1 activation, as evidenced by the phosphorylation of a substrate of mTORC1, such as p70 S6 kinase (Fig. 8A). The treatment with the mTORC1 inhibitor, rapamycin, markedly suppressed the stabilization and transcriptional activation of p53, which was demonstrated by its phosphorylation at Ser15 and the expression of p21 (Fig. 8A,B). mTORC1 requires the Raptor protein for its activation [40]. Therefore, we silenced the expression of Raptor by siRNAs, Raptor#1, and Raptor#2 and found that the knockdown of Raptor caused the inhibition of p53 activation induced by the EBP2 knockdown using siRNAs, EBP2#1, and EBP2#2 (Fig. 9A,B). However, siRNAs against Raptor failed to affect the activation of Akt induced by the EBP2 knockdown. We concluded that the EBP2 knockdown caused the activation of p53, and this appeared to be due to the activation of two types of protein kinases, Akt and mTORC1. However, these events may have occurred independently.

### 3.7 EBP2 negatively regulates p53 activity *via* the Akt/mTORC1 signaling pathway in the nucleolus of human ALCL‐derived Ki‐JK cells

**Fig. 10:**
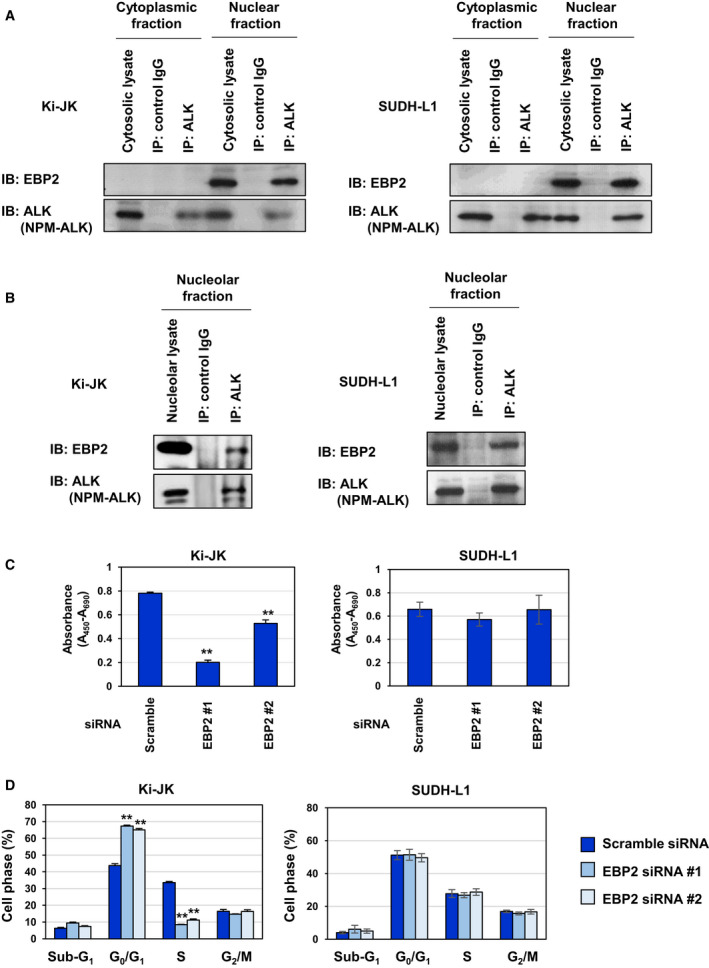
Nucleolar NPM‐ALK interacts with EBP2 and knockdown of EBP2 induced G_0_/G_1_‐phase cell cycle arrest in ALCL patient‐derived Ki‐JK cells. (A) Cytoplasmic and nuclear fractions from Ki‐JK cells and SUDH‐L1 cells were prepared and immunoprecipitated with control IgG or an anti‐ALK antibody. These prepared samples were analyzed by immunoblotting with an anti‐EBP2 or anti‐ALK antibody. (B) A nucleolar fraction was prepared from Ki‐JK cells and SUDH‐L1, and immunoprecipitated with control IgG or an anti‐ALK antibody. The nucleolar lysates and immunoprecipitates were immunoblotted with an anti‐EBP2 or anti‐ALK antibody. (C) Ki‐JK cells and SUDH‐L1 cells were transfected with scrambled siRNA and two kinds of siRNA targeting EBP2 (EBP2 siRNA #1 or EBP2 siRNA #2) and then incubated for 24 h. Cells (2.5 × 10^4^ cells/100 μL) were counted and cultured for 48 h. Cell proliferation was measured using the WST‐1 assay (*n* = 4). Error bars represent the SD of the mean. **P < 0.01 significantly different from the group of Ki‐JK cells transfected with scrambled siRNA. (D) Ki‐JK cells and SUDH‐L1 cells were transfected with scrambled siRNA, EBP2 siRNA #1, or EBP2 siRNA #2, and then incubated for 72 h. Cells were fixed, treated with propidium iodide, and subjected to a flow cytometric analysis. The ratios of cells in the G_0_/G_1_phase, S phase, and G_2_/M phase were graphed. Data were expressed as the mean ± SD (*n* = 3). **P < 0.01 significantly different from the group of Ki‐JK cells transfected with scrambled siRNA.

**Fig. 11:**
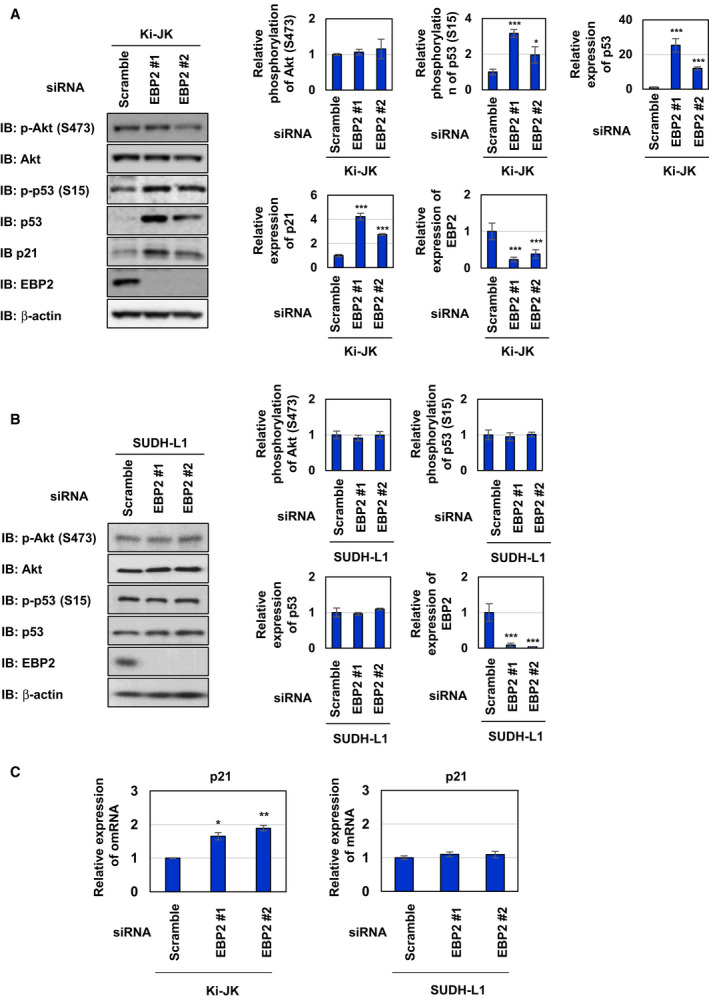
Knockdown of EBP2 activates p53 in ALCL patient‐derived Ki‐JK cells. Ki‐JK cells and SUDH‐L1 cells were transfected with scrambled siRNA, EBP2 siRNA #1, or EBP2 siRNA #2, and then incubated for 72 h. (A, B) Whole‐cell lysates were immunoblotted with an anti‐phospho‐Akt (Ser473), anti‐Akt, anti‐phospho‐p53 (Ser15), anti‐p53, anti‐p21, anti‐EBP2, or anti‐β‐actin antibody. The relative phosphorylation or expression levels of Akt, p53, p21, and EBP2 are shown in the graphs. Results represent the mean ± SD of three independent experiments. *P < 0.05, ***P < 0.001 significantly different from the control group of Ki‐JK cells or SUDH‐L1 cells transfected with scrambled siRNA. (C) Total RNA was prepared and the expression of*p21*mRNA was analyzed by quantitative real‐time PCR (*n* = 3).*Rpl13a*mRNA was analyzed as an internal control. Error bars represent the SD of the mean. *P < 0.05; **P < 0.01 significantly different from the group of Ki‐JK cells transfected with scrambled siRNA.

**Fig. 12:**
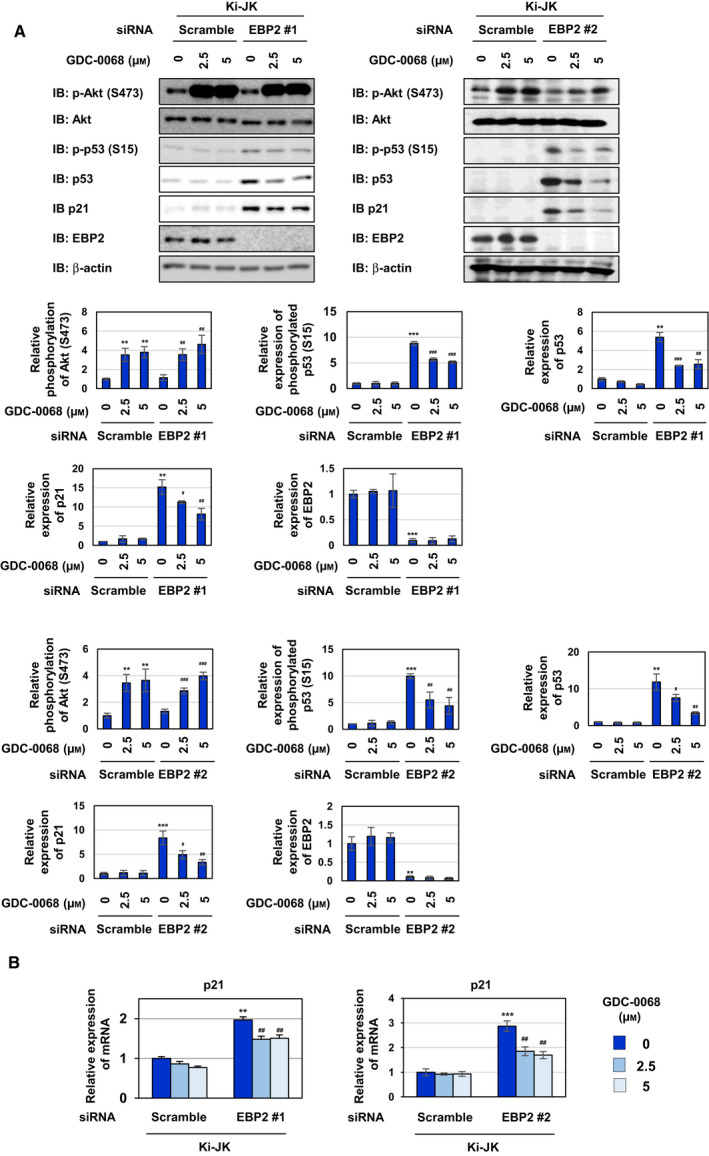
Knockdown of EBP2 activates p53 through Akt in ALCL patient‐derived Ki‐JK cells. Ki‐JK cells were transfected with scrambled siRNA, EBP2 siRNA #1, or EBP2 siRNA #2. Twenty‐four hours after transfection, cells were treated with GDC‐0068 (2.5 and 5 μm) for 48 h. (A) Whole‐cell lysates were immunoblotted with an anti‐phospho‐Akt (Ser473), anti‐Akt, anti‐phospho‐p53 (Ser15), anti‐p53, anti‐p21, anti‐EBP2, or anti‐β‐actin antibody. The relative phosphorylation or expression levels of Akt, p53, p21 and EBP2 are shown in the graphs. Results represent the mean ± SD of three independent experiments. **P < 0.01, ***P < 0.001 significantly different from the control group of Ki‐JK cells transfected with scrambled siRNA.^#^ *P* < 0.05,^##^ *P* < 0.01,^###^ *P* < 0.001 significantly different from the control group of Ki‐JK cells transfected with EBP2 siRNA #1 or EBP2 siRNA #2. (B) Total RNA was prepared, and the expression of*p21*mRNA was analyzed by quantitative real‐time PCR (*n* = 3).*Rpl13a*mRNA was analyzed as an internal control. Error bars represent the SD of the mean. **P < 0.01, ***P < 0.001 significantly different from the group of Ki‐JK cells transfected with scrambled siRNA.^##^ *P* < 0.01 significantly different from the group of Ba/F3‐NPM‐ALK transfected with EBP2 siRNA #1 or EBP2 siRNA #2.

**Fig. 13:**
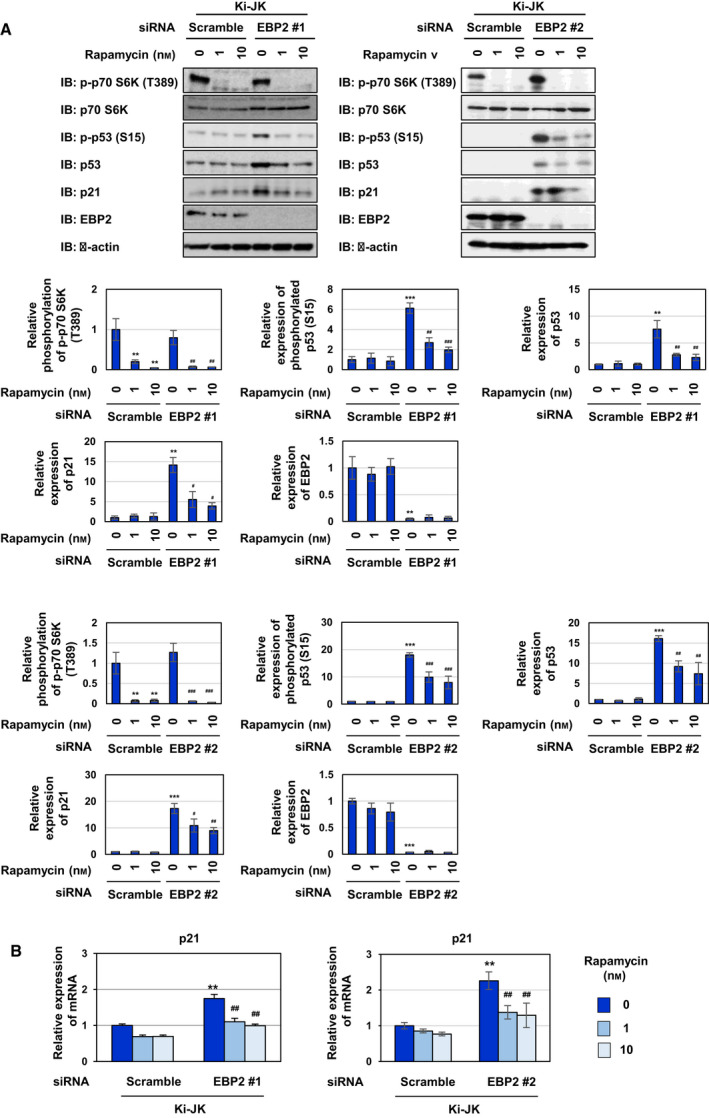
Knockdown of EBP2 activates p53 through mTORC1 pathway in ALCL patient‐derived Ki‐JK cells. Ki‐JK cells were transfected with scrambled siRNA, EBP2 siRNA #1, or EBP2 siRNA #2. Twenty‐four hours after transfection, cells were treated with rapamycin (1 and 10 nm) for 48 h. (A) Whole‐cell lysates were immunoblotted with an anti‐phospho‐p70 S6 kinase (Thr389), anti‐p70 S6 kinase, anti‐phospho‐p53 (Ser15), anti‐p53, anti‐p21, anti‐EBP2, or anti‐β‐actin antibody. The relative phosphorylation or expression levels of p70 S6 kinase, p53, p21, and EBP2 are shown in the graphs. Results represent the mean ± SD of three independent experiments. **P < 0.01, ***P < 0.001 significantly different from the control group of Ki‐JK cells transfected with scrambled siRNA.^#^ *P* < 0.05,^##^ *P* < 0.01,^###^ *P* < 0.001 significantly different from the control group of Ki‐JK cells transfected with EBP2 siRNA #1 or EBP2 siRNA #2. (B) Total RNA was prepared and the expression of*p21*mRNA was analyzed by quantitative real‐time PCR (*n* = 3).*Rpl13a*mRNA was analyzed as an internal control. Error bars represent the SD of the mean. **P < 0.01 significantly different from the group of Ki‐JK cells transfected with scrambled siRNA.^##^ *P* < 0.01 significantly different from the group of Ba/F3‐NPM‐ALK transfected with EBP2 siRNA #1 or EBP2 siRNA #2.

Ki‐JK cells and SUDH‐L1 cells are derived from a NPM‐ALK‐positive ALCL patients. It was reported that p53 gene is intact in Ki‐JK cells, whereas SUDH‐L1 cells harbor the mutation in p53 gene, which inactivates the encoded p53 tumor suppressor [41, 42]. We investigated whether the regulatory system in which NPM‐ALK induces p53 inactivation by interacting with EBP2 also functions in Ki‐JK cells and SUDH‐L1 cells. We first examined the interaction between NPM‐ALK and EBP2 in cytoplasmic and nuclear fractions of these cell lines by a co‐immunoprecipitation assay using an anti‐ALK antibody. In these cell lines, NPM‐ALK was expressed in cytoplasmic and nuclear fractions and EBP2 was expressed in nuclear fraction. Endogenous NPM‐ALK interacted with EBP2 in the nucleus of Ki‐JK cells and Ki‐JK cells (Fig. 10A). We also detected the interaction between NPM‐ALK and EBP2 in the nucleolar fraction prepared from Ki‐JK cells and SUDH‐L1 cells, suggesting that NPM‐ALK interacts with EBP2 regardless of p53 status (Fig. 10B). Whereas the proliferation rate of Ki‐JK cells was significantly reduced by the knockdown of EBP2, the proliferation rate of SUDH‐L1 cells was not affected by the knockdown of EBP2 (Fig. 10C). The knockdown of EBP2 also induced G_0_/G_1_‐phase cell cycle arrest and significantly reduced the ratio of the S phase in Ki‐JK cells but not SUDH‐L1 cells (Fig. 10D). Furthermore, the knockdown of EBP2 caused the accumulation and phosphorylation of p53 as well as the expression of p21 in Ki‐JK cells but did not alter the expression and phosphorylation levels of p53 in SUDH‐L1 cells (Fig. 11A–C). However, the activation of Akt caused by the knockdown of EBP2 was not observed in SUDH‐L1 cells and Ki‐JK cells (Fig. 11A,B). Furthermore, the EBP2 knockdown‐induced accumulation of p53 and expression of p21 were significantly inhibited by the treatment with GDC‐0068 and rapamycin in Ki‐JK cells (Figs 12A,B and 13A,B). These results suggest that the activation of Akt and mTORC1 was not triggered by the EBP2 knockdown; however, these protein kinases appear to have essential roles in ribosomal stress‐caused p53 activation in ALCL‐derived cells. Although the underlying mechanisms remain unclear, the silencing the expression of EBP2 has potential as a treatment for ALCL patients carrying the wild‐type p53 gene.

## 4 Discussion

**Fig. 14:**
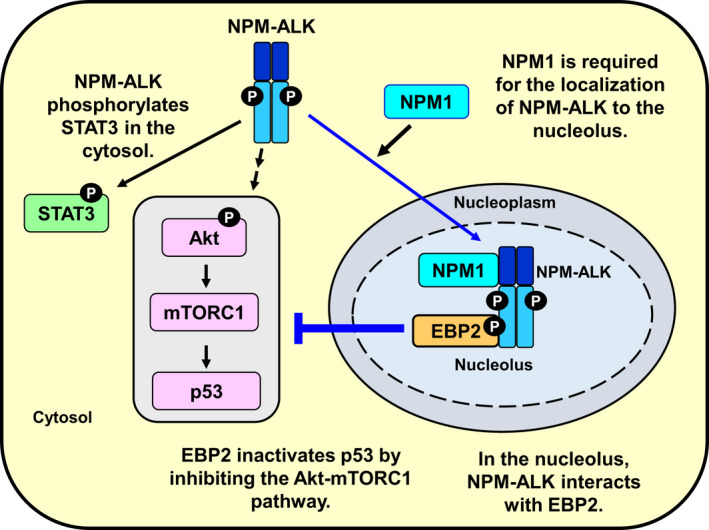
NPM‐ALK inactivates p53 by negatively regulating the activation of the Akt‐mTORC1 pathway through an interaction with EBP2 in the nucleolus. NPM‐ALK is localized in the cytosol, nucleoplasm, and nucleolus. NPM‐ALK interacts with NPM1 in a manner that is dependent on its kinase activity and is localized in the nucleolus. In the nucleolus, NPM‐ALK interacts with EBP2, which is partially involved in NPM‐ALK‐induced 28S rRNA biogenesis. Furthermore, NPM‐ALK negatively regulates p53 activity through the Akt‐mTORC1 pathway by interacting with EBP2.

The t(2;5)(p23;q35) translocation was discovered in the late 1980s [43], and several groups have since reported critical signaling pathways for NPM‐ALK‐induced oncogenesis, such as STAT3, PI3K/Akt, Ras‐ERK, and the subcellular localization of NPM‐ALK [8, 9, 10, 11]. In 1998, Mason *et al*. first reported that NPM‐ALK localized to the nucleolus [12]. However, another ALK‐fusion oncogenic protein produced by t(1;2)(q31;p23) was mainly localized to the cytosol, but not the nucleolus, and exhibited potent transforming activity. Therefore, Mason and colleagues indicated that the nucleolar accumulation of NPM‐ALK was not necessary for its oncogenic function. In the present study, by analyzing the functions of NPM‐ALK in the nucleolus, we demonstrated for the first time that NPM‐ALK in the nucleus contributed to cell proliferation through the negative regulation of p53 activity by its binding protein, EBP2 (Fig. 14).

We showed that NPM‐ALK localized to the nucleolus by binding with NPM1, a shuttling protein between the nucleus and cytoplasm (Fig. 2). Importantly, the kinase activity of NPM‐ALK was necessary for the interaction with NPM1, and the localization of NPM‐ALK into the nucleolus (Fig. 2). The oligomerization of NPM1 is facilitated by the phosphorylation of NPM1 at Ser125, which is a substrate of IKKα [44]. Although there is currently no evidence to show that NPM‐ALK induces the activation of the IKK‐NF‐κB pathway, the phosphorylation of NPM1 may be mediated by IKKα activated through the complex signaling pathway caused by NPM‐ALK. Ceccon *et al*. also demonstrated that NPM1 is required for the localization of NPM‐ALK to the nucleus using NPM^−/−^/p53^−/−^ MEFs and p53^−/−^ MEFs and showed that expression of NPM‐ALK induced apoptosis through causing DNA damage in NPM^−/−^/p53^−/−^ MEFs but not p53^−/−^ MEFs [15]. However, we observed that the viability of NPM^−/−^/p53^−/−^ MEFs was not altered by enforced expression of NPM‐ALK. The difference in this phenomenon may depend on the degree of the expressed NPM‐ALK. Ceccon *et al*. also showed that enforced expression of NPM‐ALK in the nucleus did not transform cells using a fusion NPM‐ALK construct containing the total NPM1 protein which is localized entirely to the nucleus. However, the characteristics of this artificially constructed NPM‐ALK are totally different from those of the detected NPM‐ALK in ALCL patient, and it is insufficient to clarify the function of NPM‐ALK in the nucleus.

We also found that NPM‐ALK interacted with EBP2 in the nucleolus. EBP2 has been identified as a binding partner of Epstein–Barr virus (EBV) nuclear antigen 1, which plays a critical role in EBV segregation [45, 46]. EBP2 is also reported to be involved in preribosomal RNA processing and ribosomal subunit assembly [28, 47]. We showed that the knockdown of EBP2 partially suppressed the NPM‐ALK‐induced biogenesis of 28S rRNA and effectively activated p53 (Figs 4 and 5). Previous studies reported that actinomycin D induced the activation of several protein kinases, such as Akt and mTORC1, leading to p53 activation [48, 49]. However, compared to these findings, the effects of EBP2 knockdown on NPM‐ALK‐accelerated rRNA synthesis were very weak; therefore, the knockdown of EBP2 may activate p53 activation by other mechanisms. On the other hand, the knockdown of DDX21, which is another interactor with NPM‐ALK, had no effect on NPM‐ALK‐induced RNA biogenesis and cell proliferation (Figs 4 and 5). Although we could not reveal the function of DDX21 in the current study, it is important to clarify the function of DDX21 in NPM‐ALK‐induced tumorigenesis.

The phosphorylation of Akt at Thr308 was constitutively detected in Ba/F3‐NPM‐ALK, and the EBP2 knockdown significantly increased the phosphorylation of Akt at Ser473 (Fig. 6). In contrast, the phosphorylation of Akt at Ser473 was not enhanced by the EBP2 knockdown in human Ki‐JK cells (Fig. 11). Although we currently cannot explain this discrepancy, Akt clearly plays a critical role in EBP knockdown‐induced p53 activation. The phosphorylation of Akt at Ser473 was reported to be caused by various protein kinases, such as mTORC2, PI3K, DNA‐PK, and ATM [50, 51]. LY294002 inhibits PI3K and PI3K‐related kinases including mTOR and DNA‐PK [52] and inhibited the enhanced activation of Akt and reduced the accumulation of p53 caused by the knockdown of EBP2 in Ba/F3‐NPM‐ALK in the present study (Fig. S5). The knockdown of EBP2 failed to induce DNA damage, as evidenced by the phosphorylation of γ‐H2AX in Ba/F3‐NPM‐ALK (Fig. S4), suggesting that the DNA damage‐dependent pathway including DNA‐PK, ATM, and ATR is dispensable for EBP2 knockdown‐induced p53 activation.

A previous study reported that myristoylated constitutively active AKT (myr‐Akt) induced p53 activation through mTORC1 and the nucleolar sequestration of Mdm2 [53]. Furthermore, the loss of PTEN, a competitor for PI3K, induced the activation of p53 through mTORC1 and mTORC2 [54]. In the PTEN‐deficient background, the binding activity of p53 to mTORC1 and mTORC2 was enhanced, whereas that to Mdm2 was weakened. The knockdown of EBP2 increased the expression of Mdm2 in Ba/F3‐NPM‐ALK and Ki‐JK cells (Fig. S6A,B), which may have been caused by the activation of p53 since the *mdm2* gene is a target of p53 [55]. The knockdown of EBP2 increased the expression of Mdm2 in the cytosol and decreased the expression of Mdm2 in the nucleolus (Fig. S6C). On the other hand, the interaction between Mdm2 and p53 was not altered by the knockdown of EBP2 in Ba/F3‐NPM‐ALK (Fig. S6D). A previous study also reported that the Akt‐mediated phosphorylation of Mdm2 at Ser166 and Ser186 promotes its nuclear translocation, resulting in the ubiquitination and degradation of p53 [56]. This report suggests that the increases the expression of Mdm2 in cytosol and the decreases in nucleolus caused by EBP2 knockdown may inhibit Mdm2‐induced p53 degradation, leading to p53 accumulation. Although further experiments are needed, the present results appear to provide support for the requirement of Akt for EBP2 knockdown‐induced p53 activation.

It is still unclear whether the interaction of EBP2 with NPM‐ALK is indispensable for the promotion of cell proliferation by NPM‐ALK. To examine the importance of the interaction of NPN‐ALK with EBP2 in NPM‐ALK‐induced cell proliferation, it would be necessary to perform the genome editing to express EBP2 mutants lacking the ability to bind to NPM‐ALK. Although it was shown that phosphorylation level of NPM‐ALK in nucleoplasm and nucleolus was lower than that of NPM‐ALK in cytosol (Fig. 1), we found that the tyrosine phosphorylation of EBP2 was induced in a manner that depended on the kinase activity of NPM‐ALK (Fig. 3). These results suggest the possibilities that EBP2 is directly phosphorylated by NPM‐ALK in nucleolus although its catalytic activity was not high and that EBP2 is phosphorylated by some tyrosine kinase which is activated by NPM‐ALK in nucleolus. Although its biological significance currently remains unknown, two tyrosine residues Tyr136 and Tyr265 in EBP2 were previously shown to be phosphorylated in large phosphoproteomic studies [57]. It is important to investigate whether EBP2 is a substrate of NPM‐ALK, and further studies are needed to clarify the roles of the phosphorylation of EBP2 in the NPM‐ALK‐induced inactivation of p53 through the Akt‐mTORC1 pathway by utilizing EBP2 mutants in which tyrosine residues are substituted.

The activity of p53 is often inactivated by TP53 mutations or oncoproteins‐induced suppressive mechanisms in cancer cells. However, the majority of ALK‐positive ALCL patients carry the wild‐type p53 gene [58]. Cui *et al*. [59] showed that NPM‐ALK suppresses the activation of p53 in JNK and MDM2‐dependent manners using pharmacological inhibitors. In addition to these findings, the present results revealed a novel repressive mechanism of p53 activity in NPM‐ALK‐expressing cells by EBP2 and its potential therapeutic strategies for ALCL by utilizing nucleolar stress.

## 5 Conclusions

In the current study, we focused the mechanism how an oncogenic NPM‐ALK localizes to the nucleolus and the function of nucleolar NPM‐ALK. NPM‐ALK localizes to the nucleolus by binding to NPM1 in a manner that is dependent on its kinase activity. In the nucleolus, NPM‐ALK interacts with EBP2, which is involved in rRNA biosynthesis. Strikingly, silencing the expression of EBP2 caused the activation of p53 tumor suppressor through the Akt‐mTORC1 pathway in ALCL‐derived cells. Our finding suggests the possibility that EBP2 would be a novel therapeutic target for ALCL.

## Conflict of interest

The authors declare no conflict of interest.

## Author contributions

YU and MF‐T performed all of the experiments except LC‐MS. KT performed LC‐MS and contributed to preparation of retrovirus. YU, KT, and HT analyzed data. YU, KY, and MF‐T wrote the manuscript.

## Supporting information


**Fig. S1.** The expression of NPM1 has no effect on the phosphorylation of NPM‐ALK or STAT3 in NPM1^−/−^/p53^−/−^ MEF.
**Fig. S2.** The treatment with the protein‐tyrosine phosphatase inhibitor pervanadate enhances tyrosine phosphorylation levels in transduced Ba/F3 cells.
**Fig. S3.** EBP2 knockdown has no effect on the subcellular localization of ribosomal proteins in Ba/F3 cells expressing NPM‐ALK.
**Fig. S4.** EBP2 knockdown does not induce DNA damage in Ba/F3 cells expressing NPM‐ALK.
**Fig. S5.** LY294002 inhibits the phosphorylation of Akt, accumulation of p53, and expression of p21 induced by the knockdown of EBP2 in Ba/F3 cells expressing NPM‐ALK.
**Fig. S6.** EBP2 knockdown have no effects on the nucleolar sequestration of mdm2 or the interaction between p53 and mdm2 in Ba/F3 cells expressing NPM‐ALK.
