# Supplementary material for: EBP2, a novel NPM‐ALK‐interacting protein in the nucleolus, contributes to the proliferation of ALCL cells by regulating tumor suppressor p53
Source: Mol Oncol. 2020 Nov 19;15(1):167–94. doi: 10.1002/1878-0261.12822 (PMC7782078; doi:10.1002/1878-0261.12822)
Supplement: Supplementary file 1 — Fig. S1. The expression of NPM1 has no effect on the phosphorylation of NPM‐ALK or STAT3 in NPM1−/−/p53−/− MEF. Fig. S2. The treatment with the protein‐tyrosine phosphatase inhibitor pervanadate enhances tyrosine phosphorylation levels in transduced Ba/F3 cells. Fig. S3. EBP2 knockdown has no effect on the subcellular localization of ribosomal proteins in Ba/F3 cells expressing NPM‐ALK. Fig. S4. EBP2 knockdown does not induce DNA damage in Ba/F3 cells expressing NPM‐ALK. Fig. S5. LY294002 inhibits the phosphorylation of Akt, accumulation of p53, and expression of p21 induced by the knockdown of EBP2 in Ba/F3 cells expressing NPM‐ALK. Fig. S6. EBP2 knockdown have no effects on the nucleolar sequestration of mdm2 or the interaction between p53 and mdm2 in Ba/F3 cells expressing NPM‐ALK. [file MOL2-15-167-s001.pdf]

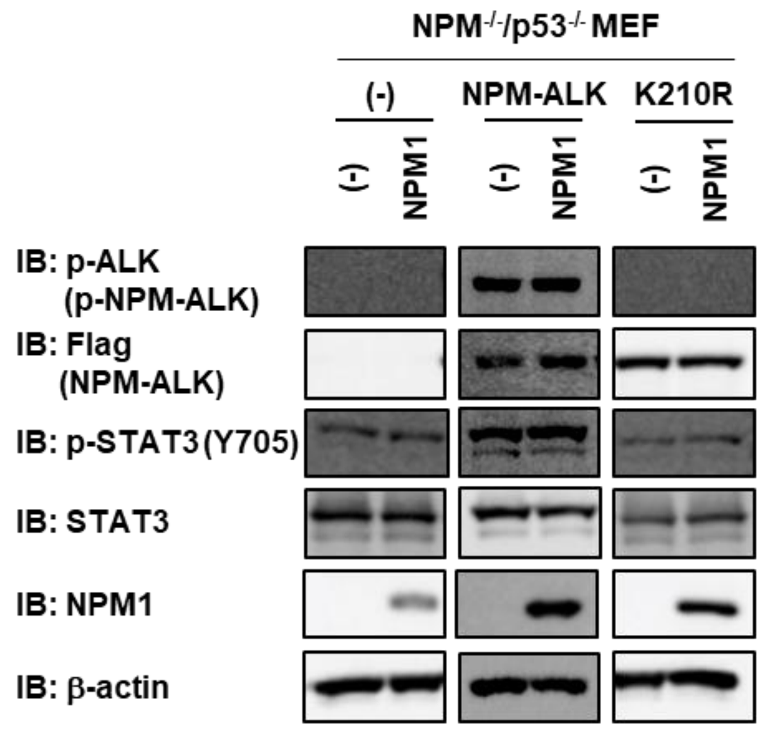

Fig. S1 The expression of NPM1 has no effect on the phosphorylation of NPM-ALK or STAT3 in $NPM1^{-/-}/p53^{-/-}$ MEF.

$NPM1^{-/-}/p53^{-/-}$ MEF and $NPM1^{-/-}/p53^{-/-}$ MEF expressing NPM-ALK and $NPM1^{-/-}/p53^{-/-}$ MEF expressing the kinase dead mutant of NPM-ALK K210R were infected with an empty virus (-) and expressed NPM1 by retroviral infection. Whole cell lysates from transduced MEF were prepared and immunoblotted with an anti-phospho-ALK (Tyr1604), anti-Flag, anti-phospho-STAT3 (Tyr705), anti-STAT3, anti-NPM1, or anti-β-actin antibody.

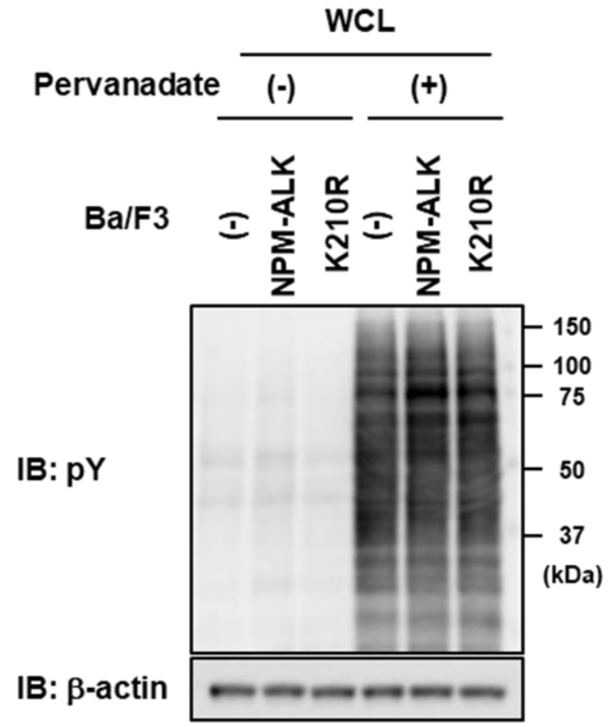

Fig. S2 The treatment with the protein-tyrosine phosphatase inhibitor pervanadate enhances tyrosine phosphorylation levels in transduced Ba/F3 cells.

Ba/F3 cells infected with an empty vector (-) and Ba/F3 cells expressing NPM-ALK and its kinase dead mutant K210R were treated with 0.5 mM pervanadate for 30 min. Whole cell lysates were immunoblotted with an anti-phospho-tyrosine or anti-β-actin antibody.

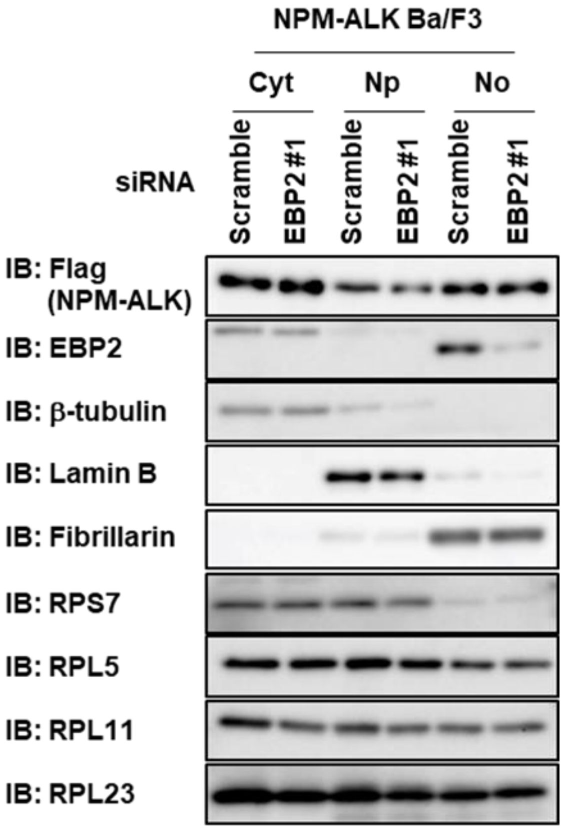

Fig. S3 EBP2 knockdown has no effect on the subcellular localization of ribosomal proteins in Ba/F3 cells expressing NPM-ALK.

Ba/F3 cells expressing NPM-ALK were transfected with scramble siRNA and EBP2 siRNA #1. Twenty hours after transfection, cytosolic, nucleoplasmic, and nucleolar fractions of cells were prepared and immunoblotted with an anti-Flag, anti-EBP2, anti-β-tubulin, anti-Lamin B, anti-Fibrillarin, anti-RPS7, anti-RPL5, anti-RPL11, or anti-RPL23 antibody.

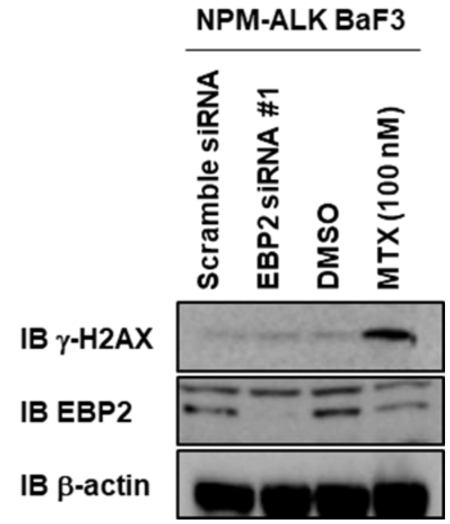

Fig. S4 EBP2 knockdown does not induce DNA damage in Ba/F3 cells expressing NPM-ALK.

Ba/F3 cells expressing NPM-ALK were transfected with scrambled siRNA or EBP2 siRNA #1 and then incubated for 20 h. Ba/F3 cells expressing NPM-ALK were treated with methotrexate (100 nM) for 24 h. Methotrexate was used as a positive control that induces DNA damage. Whole cell lysates were immunoblotted with an anti-phospho-Histone H2AX (Ser139), anti-EBP2, or anti-β-actin antibody.

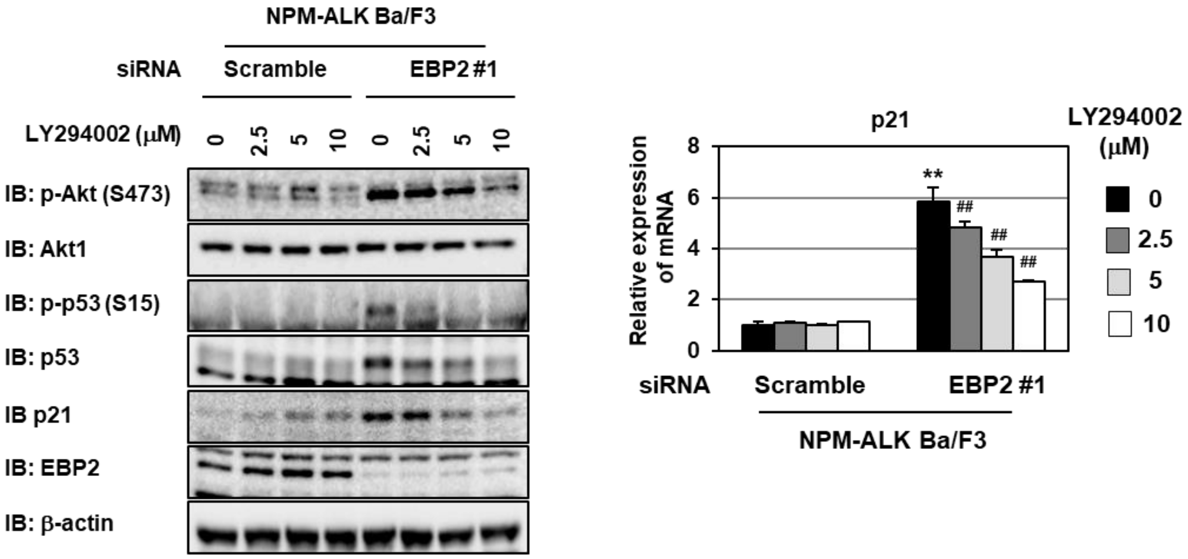

Fig. S5 LY294002 inhibits the phosphorylation of Akt, accumulation of p53, and expression of p21 induced by the knockdown of EBP2 in Ba/F3 cells expressing NPM-ALK.

Ba/F3 cells were transfected with scramble siRNA and EBP2 siRNA #1. Fourteen hours after transfection, cells were treated with LY294002 (2.5, 5, and 10 μM) for 6 h. (A) Whole cell lysates were immunoblotted with an anti-phospho-Akt (Ser473), anti-Akt, anti-phospho-p53 (Ser15), anti-p53, anti-p21, anti-EBP2, or anti-β-actin antibody. (B) Total RNA was prepared and the expression of *p21* mRNA was analyzed by quantitative real-time PCR (n = 3). *Rpl13a* mRNA was analyzed as an internal control. Error bars represent the SD of the mean. $^{**}P < 0.01$ significantly different from the group of Ba/F3 cells expressing NPM-ALK transfected with scrambled siRNA. $^{\#\#}P < 0.01$ significantly different from the group of Ba/F3 cells expressing NPM-ALK transfected with EBP2 siRNA #1.

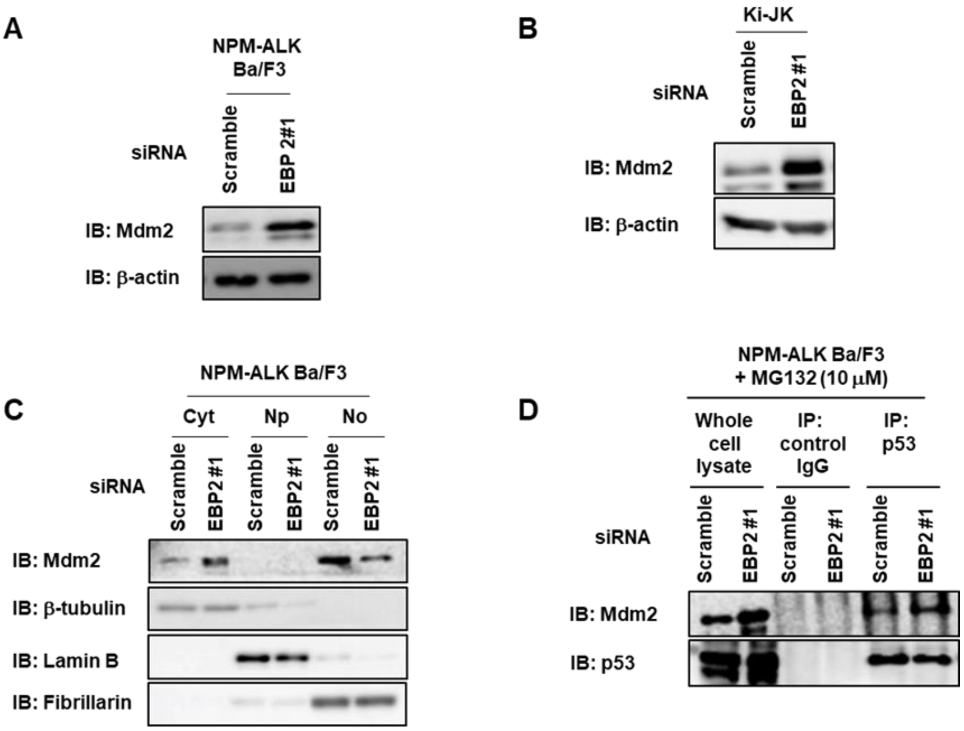

Fig. S6. EBP2 knockdown have no effects on the nucleolar sequestration of mdm2 or the interaction between p53 and mdm2 in Ba/F3 cells expressing NPM-ALK.

Ba/F3 cells expressing NPM-ALK (A, C, D) and Ki-JK cells (B) were transfected with scrambled siRNA or EBP2 siRNA #1 and then incubated for 20 h. (A) Whole cell lysates were immunoblotted with an anti-mdm2 or anti-β-actin antibody. The same blot (β-actin) used in Fig. 5B was shown here. (B) Whole cell lysates prepared from Ki-JK were immunoblotted with an anti-mdm2 or anti-β-actin antibody. The same blot (β-actin) used in Fig. 7D was shown here. (C) Cytosolic, nucleoplasmic, and nucleolar fractions of cells were prepared and immunoblotted with an anti-mdm2, anti-β-tubulin, anti-Lamin B, or anti-Fibrillarin antibody. The same blots (β-tubulin, Lamin B, and Fibrillarin) used in Fig. 3S were shown here. (D) Ba/F3 cells expressing NPM-ALK were transfected with scrambled siRNA or EBP2 siRNA #1. Fourteen hours after transfection, cells were treated with the proteasome inhibitor, MG132 (10 μM) for 6 h. Cell lysates were immunoprecipitated with control IgG or an anti-p53 antibody and then immunoblotted with an anti-mdm2 or anti-p53 antibody. A light chain-specific secondary antibody was used to detect p53.
